# The aspirin-induced long non-coding RNA OLA1P2 blocks phosphorylated STAT3 homodimer formation

**DOI:** 10.1186/s13059-016-0892-5

**Published:** 2016-02-22

**Authors:** Haiyan Guo, Jun Liu, Qiwen Ben, Yuehong Qu, Man Li, Ying Wang, Wantao Chen, Jianjun Zhang

**Affiliations:** Department of Clinical Laboratory, Ninth People’s Hospital, Shanghai Jiao Tong University School of Medicine, 280 Mohe Road, Shanghai, 201999 PR China; Department of Oral & Maxillofacial-Head Neck Oncology, Ninth People’s Hospital, Shanghai Jiao Tong University School of Medicine, 639 Zhizaoju Road, Shanghai, 200011 PR China; Department of Integrative Medicine, Zhongshan Hospital, Fudan University, Shanghai, PR China; Department of Gastroenterology, Ruijin Hospital, Shanghai Jiao Tong University School of Medicine, Shanghai, PR China

**Keywords:** Aspirin, FOXD3, STAT3, lncRNA OLA1P2, Cancer

## Abstract

**Background:**

Although the chemopreventive effects of aspirin have been extensively investigated, the roles of many cell components, such as long non-coding RNAs, in these effects are still not completely understood.

**Results:**

We identify an aspirin-induced upregulated lncRNA, OLA1P2, in human colorectal cancer. Aspirin induces demethylation of the FOXD3 promoter and promotes expression of the FOXD3 gene. Subsequently, upregulated FOXD3 protein transcriptionally activates lncRNA OLA1P2 expression. OLA1P2 upregulation markedly affects STAT3 signaling pathway activity by inhibiting the nuclear import of phosphorylated STAT3. The phosphorylation of tyrosine-705 of STAT3 is the first step in OLA1P2 binding, and the formation of phosphorylated STAT3 homodimers is subsequently blocked. OLA1P2 interacts directly with STAT3 due to OLA1P2 sharing the same conservative STAT3 transcription response element as STAT3 targets. Regular use of aspirin dramatically decreases the number of metastatic nodules of cancer cells in immunodeficient mouse lungs, and OLA1P2 silencing markedly weakens the anti-metastatic activity of aspirin in the lungs. Additionally, low OLA1P2 levels are associated with malignant transformation and lower overall survival in cancers.

**Conclusions:**

The present study finds that the aspirin-FOXD3-OLA1P2-STAT3 axis exhibits exciting anticancer effects and provides new insights into the chemopreventive mechanisms underlying aspirin use.

**Electronic supplementary material:**

The online version of this article (doi:10.1186/s13059-016-0892-5) contains supplementary material, which is available to authorized users.

## Background

Multiple randomized controlled trials have demonstrated that aspirin can protect against different types of cancer, particularly colorectal cancer (CRC) [1]. Daily use of aspirin at moderate doses markedly reduces the risk of CRC and is associated with decreased risk of new CRC in patients with a personal history of CRC [2, 3]. Even the lowest daily dose of aspirin was associated with a reduced risk of CRC [4, 5]. The effects of aspirin on inhibiting COX-1-dependent platelet function represent an important mechanism for preventing carcinogenesis [6]. Additionally, by inhibiting PP2A enzymatic activity, aspirin causes the degradation of phosphorylated β-catenin [7]. Aspirin may act through inhibition of the Ras/c-Raf interaction and upregulation of MKPs to suppress ERK-mediated signaling [8]. Inhibition of NF-kappaB activity by aspirin could make human colon cancer cells more susceptible to apoptosis [9]. Aspirin also reduces mTOR signaling by inhibiting the mTOR effectors S6K1 and 4E-BP1 in CRC [10]. Although the anticancer molecular mechanisms underlying the effects of aspirin have been extensively investigated, the involvement of a large number of cell components, such as long non-coding RNAs, is still not completely understood.

Long non-coding RNAs (lncRNAs) have emerged as key regulators involved in controlling fundamental biological processes [11]. lncRNA HOTAIR expression is associated with genome-wide reprogramming of PRC2 function in CRC, in which upregulation of HOTAIR is a critical element in metastatic progression [12]. A high expression level of the lncRNA-CLMAT3 is significantly associated with liver metastasis of CRC and is an independent prognostic indicator of survival for patients with CRC [13]. The lncRNA MYCLos could function in CRC cell proliferation by regulating MYC target genes, such as p21 [14]. The lncRNA CCAT1-L, which is transcribed specifically in human CRC, plays a key role in the transcriptional regulation of MYC [15]. The lncRNA MALAT1 could promote cell proliferation and metastasis in CRC by binding to SFPQ and releasing the proto-oncogene PTBP2 from the SFPQ/PTBP2 complex [16]. The lncRNA CCAT2 is highly overexpressed in CRC, and this lncRNA promotes tumor growth, metastasis, and chromosomal instability through TCF7 L2-mediated transcriptional regulation [17]. However, little is known about the regulation and functions of lncRNAs in the treatment of CRC with aspirin.

In this study, we investigated global lncRNA expression profiles using microarray analysis of CRC cells treated with aspirin, and we identified the profiles of transcription factors interacting with the lncRNA OLA1P2 (HGNC: 45277) promoter via mass spectrometry. We fully analyzed the mechanisms underlying altered OLA1P2 levels and the effects on the phenotypes of CRC cells *in vitro* and *in vivo*. Our findings suggested that aspirin could suppress cancer cell growth and metastasis through the induction of lncRNA OLA1P2 transcription and the subsequent blocking of phosphorylated STAT3 (signal transducer and activator of transcription 3) homodimers formation.

## Results

### Aspirin-induced lncRNA OLA1P2 upregulation in cancer cells

**Fig. 1:**
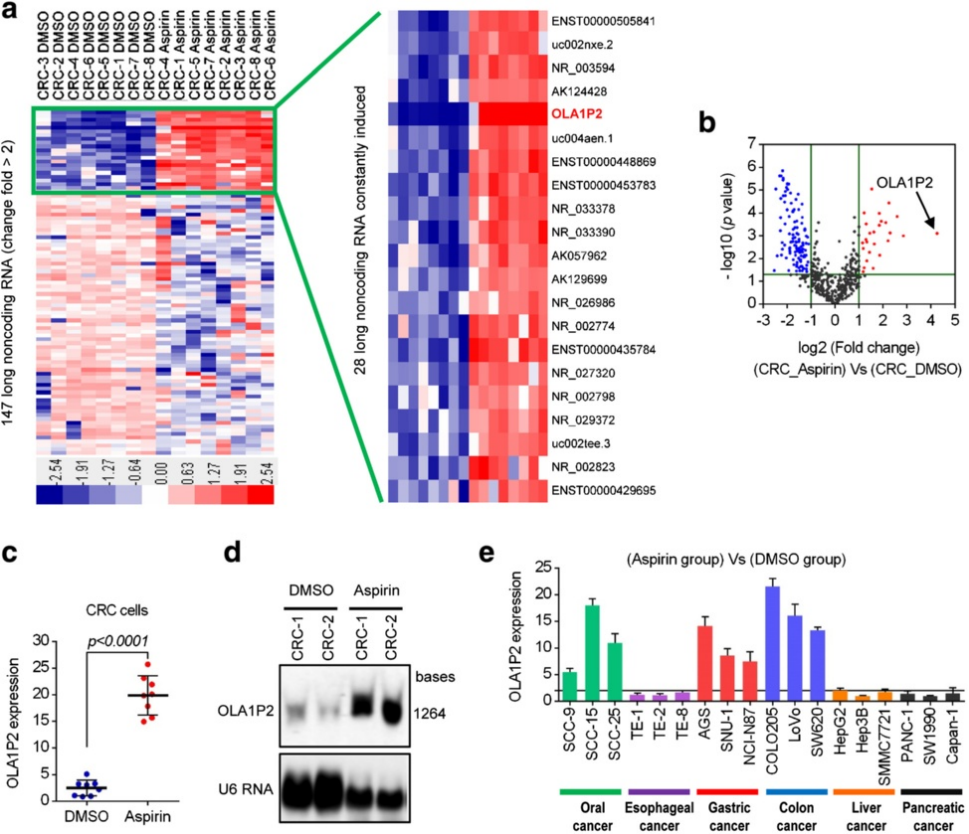
Aspirin-induced lncRNA OLA1P2 upregulation in cancer cells. **a** Primary cultured colon cancer cells were treated with aspirin (100 μM) for 48 h and then evaluated to determine their lncRNA profiles using an lncRNA expression microarray. Red indicates high expression; blue indicates low expression. **b** A volcano plot showing the relationship between the *P* values and the magnitude of the differences in the expression values of the samples in the different groups. **c** The expression level of OLA1P2 was validated by qRT-PCR analysis in all eight pairs of CRC cells with or without aspirin treatment. **d** Primary cultured colon cancer cells were treated with aspirin (100 μM) for 48 h and then evaluated for OLA1P2 expression using northern blotting analysis. **e** Cancer cell lines were treated with aspirin (100 μM) for 48 h and then evaluated for OLA1P2 expression using qRT-PCR analysis

To identify the lncRNA profiles of colorectal carcinoma (CRC) cells treated with aspirin, we performed microarray analysis using primary cultured cancer cells obtained from eight clinical CRC tissues. After the cells were treated with aspirin, 28 lncRNAs were statistically upregulated more than two-fold (Fig. 1a). OLA1P2 (HGNC: 45277) was the most changed lncRNA among these upregulated lncRNAs (fold change >20) (Fig. 1b). We validated the expression level of OLA1P2 by qRT-PCR analysis in all eight pairs of CRC cells with or without aspirin treatment (Fig. 1c). Using northern blot analysis, we also identified OLA1P1 upregulation in primary cultured CRC cells treated with aspirin (Fig. 1d). Considering that many digestive system cancers could possess similar characteristics, we assessed OLA1P2 expression in other types of cancer cells. OLA1P2 expression was dramatically induced after aspirin treatment in oral cancer, gastric cancer, and colon cancer cell lines, but not in esophageal cancer, liver cancer, and pancreatic cancer cell lines (Fig. 1e).

### Aspirin promoted OLA1P2 transcription through FOXD3 upregulation

**Fig. 2:**
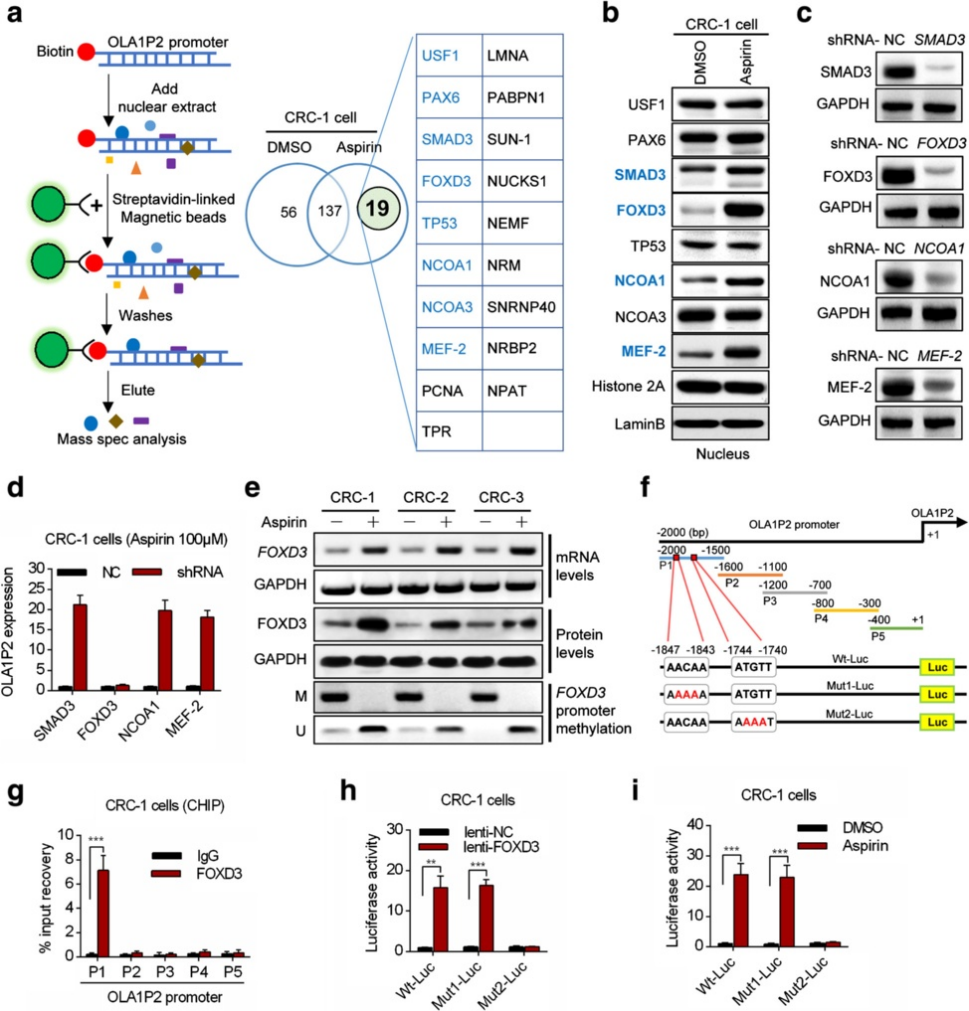
Aspirin promoted OLA1P2 transcription through FOXD3 upregulation. **a** The biotin-labeled OLA1P2 promoter was mixed with the nuclear extract separated from DMSO/aspirin-treated primary culture cancer cells. The eluted proteins were then analyzed using mass spectrometry methods. **b** The protein levels in the nuclei of primary cultured cells treated with aspirin (100 μM) for 48 h were analyzed using immunoblotting methods. **c** Protein levels in primary culture cancer cells transfected with shRNA vectors for 48 h were analyzed by immunoblot. **d** OLA1P2 expression was analyzed by qRT-PCR methods in primary culture cancer cells transfected with the shRNA vector for 48 h and then treated with aspirin (100 μM) for 48 h. **e** QRT-PCR analysis of *FOXD3* mRNA expression in CRC cells treated with aspirin (top lanes). Immunoblot analysis of FOXD3 protein levels in CRC cells treated with aspirin (middle lanes). Quantitative methylation-specific PCR analysis of *FOXD3* promoter methylation levels in CRC cells treated with aspirin (bottom lanes). M: methylation-specific primer; U: unmethylation-specific primer. **f** Diagram showing conserved FOXD3 response elements in the OLA1P2 promoter. **g** ChIP assays using anti-FOXD3 or anti-IgG antibodies were performed to determine the affinity of FOXD3 for the OLA1P2 promoter in primary culture cancer cells. **h** Primary culture cancer cells were co-transfected with the lenti-FOXD3 vector and a luciferase reporter for 48 h. **i** Primary culture cancer cells were transfected with a luciferase reporter for 12 h and were then treated with aspirin (100 μM) for 48 h. **: *P* <0.01; ***: *P* <0.001

To investigate the transcription factors that are responsible for OLA1P2 expression, we performed a pull-down experiment in the nuclear extract using the biotin-labeled OLA1P2 promoter. Nuclear proteins of CRC cells were incubated and pulled down by biotin-labeled OLA1P2 promoter. One-shot mass spectrometry analyses were then performed to analyze the purified nuclear proteins. As shown in Fig. 2a, 19 proteins were specifically identified in the aspirin-treated group, and eight of the proteins (blue-labeled) were previously confirmed to be transcription factors or nuclear receptor coactivators. Using immunoblot analysis, we found that the SMAD3, FOXD3 (forkhead box D3), NCOA1, and MEF-2 proteins were dramatically increased in the nucleus following aspirin treatment (Fig. 2b). After these transcription factors were silenced individually (Fig. 2c), only FOXD3 dysregulation obviously affected OLA1P2 expression under aspirin treatment (Fig. 2d). Both *FOXD3* mRNA and FOXD3 protein levels in the whole cell lysate of CRC cells were upregulated under aspirin treatment (Fig. 2e). In addition, aspirin-induced demethylation of the *FOXD3* promoter may be responsible for FOXD3 overexpression in CRC cells (Fig. 2e, bottom lanes). Using chromatin immunoprecipitation analysis, we showed that part 1 (P1) of the OLA1P2 promoter exhibited a strong binding affinity with the FOXD3 protein (Fig. 2f, g). We then analyzed the nucleic acid sequence in greater detail and identified two transcriptional response elements for FOXD3 in part 1 of the OLA1P2 promoter (Fig. 2f). Mutations in the putative FOXD3-binding sites (the region from -1744 to -1740 bp) rendered the luciferase constructs unresponsive to FOXD3 induction (Fig. 2h) and to aspirin treatment (Fig. 2i).

### LncRNA OLA1P2 blocked the nuclear import of phosphorylated STAT3 (Tyr705)

**Fig. 3:**
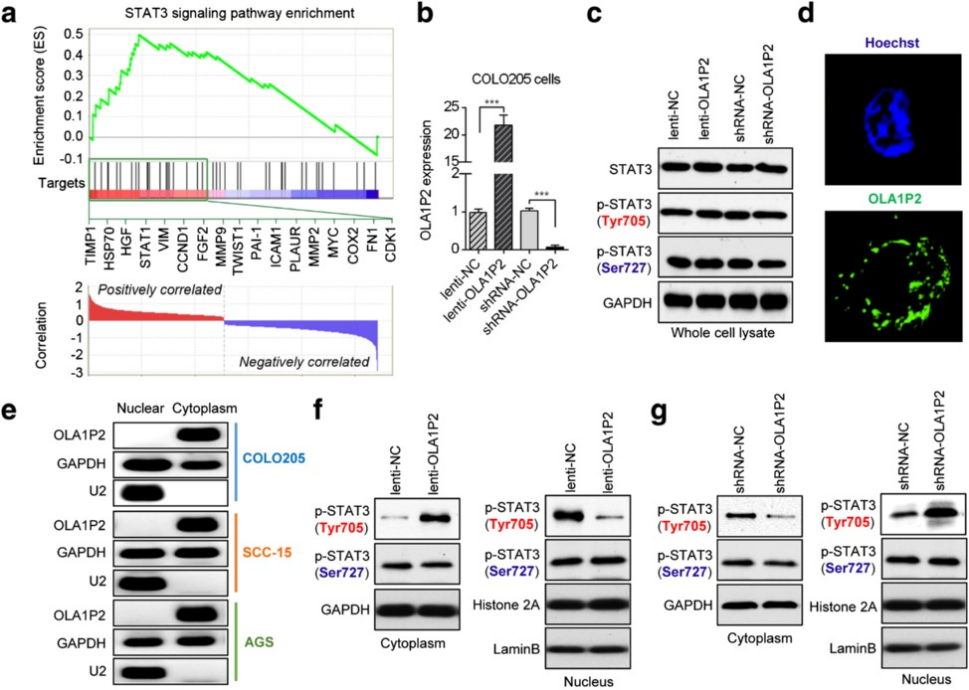
OLA1P2 affected the translocation of the phosphorylated STAT3 protein. **a** GSEA demonstrated enrichment of STAT3 target genes. The top of the panel shows the enrichment scores for genes associated with STAT3 signaling pathway targets. The black lines represent the 17 most highly correlated targets. The bottom of the panel shows the ranking scores (correlations of all genes associated with STAT3 signaling pathway targets with aspirin). **b** QRT-PCR analysis of OLA1P2 expression in the colon cancer cell line COLO205 transfected with lenti-OLA1P2, shRNA-OLA1P2, or negative controls. **c** Total STAT3 protein and phosphorylated STAT3 protein levels were analyzed by immunoblot in COLO205 cells transfected with lenti-OLA1P2 or shRNA-OLA1P2. **d** RNA FISH analysis of OLA1P2 localization in COLO205 cells. **e** QRT-PCR analysis of OLA1P2 localization. OLA1P2 was mainly present in the cytoplasm. U2 RNA was used as a positive control for nuclear RNA. **f** Phosphorylated STAT3 protein levels in the cytoplasm and nuclei of COLO205 cells transfected with lenti-OLA1P2 were analyzed by immunoblot analysis. **g** Phosphorylated STAT3 protein levels in the cytoplasm and nuclei of COLO205 cells transfected with shRNA-OLA1P2 were analyzed by immunoblot analysis. ***: *P* <0.001

To identify the targets regulated by OLA1P2, we performed a global gene expression profiling analysis in OLA1P2-silenced primary cultured cancer cells obtained from eight clinical CRC tissues. After OLA1P2 was silenced, 59 genes were statistically upregulated more than two-fold (Additional file 2: Figure S1). We conducted gene set enrichment analysis (GSEA), which is a bioinformatics method that determines whether a set of pathways shows statistically significant enrichment in the most altered genes [18]. One of the GSEA plots indicated significant enrichment of the STAT3 (signal transducer and activator of transcription 3) signaling pathway in the genes regulated by OLA1P2 (*P* <0.001) (Fig. 3a). Other related GSEA plots with low enrichment score are listed in Additional file 2: Figure S1. Using qRT-PCR analysis, we confirmed these upregulated STAT3 targets in OLA1P2-silenced cancer cell lines (Additional file 2: Figure S2). To determine whether STAT3 expression was regulated by OLA1P2, we infected cancer cells with a lentivirus expression vector (lenti-OLA1P2) or a short hairpin RNA vector (shRNA-OLA1P2) (Fig. 3b; Additional file 2: Figure S3A). Neither endogenous total STAT3 protein levels nor phosphorylated STAT3 protein levels were clearly affected by OLA1P2 (Fig. 3c; Additional file 2: Figure S3B).

To determine the precise mechanism underlying regulation of STAT3 signaling pathway activity by OLA1P2 in tumors, we examined the cellular location of OLA1P2. Using RNA FISH (fluorescent *in situ* hybridization) technology, we determined the cytoplasm localization of OLA1P2 in the COLO205 cells (Fig. 3d). By isolating both nuclear and cytoplasmic RNA, we confirmed that OLA1P2 was mainly present in the cytoplasm (Fig. 3e). To determine whether phosphorylated STAT3 protein translocation was regulated by OLA1P2, we separated this protein from the cytoplasm and nuclear extracts. The nuclear import of phosphorylated STAT3 (Tyr705) protein, but not phosphorylated STAT3 (Ser727) protein, was largely blocked when OLA1P2 was overexpressed (Fig. 3f; Additional file 2: Figure S3C). In contrast, the nuclear import of phosphorylated STAT3 (Tyr705) protein, but not phosphorylated STAT3 (Ser727) protein, was dramatically promoted when OLA1P2 was silenced (Fig. 3g; Additional file 2: Figure S3D).

### LncRNA OLA1P2 directly interacted with phosphorylated STAT3 (Tyr705)

**Fig. 4:**
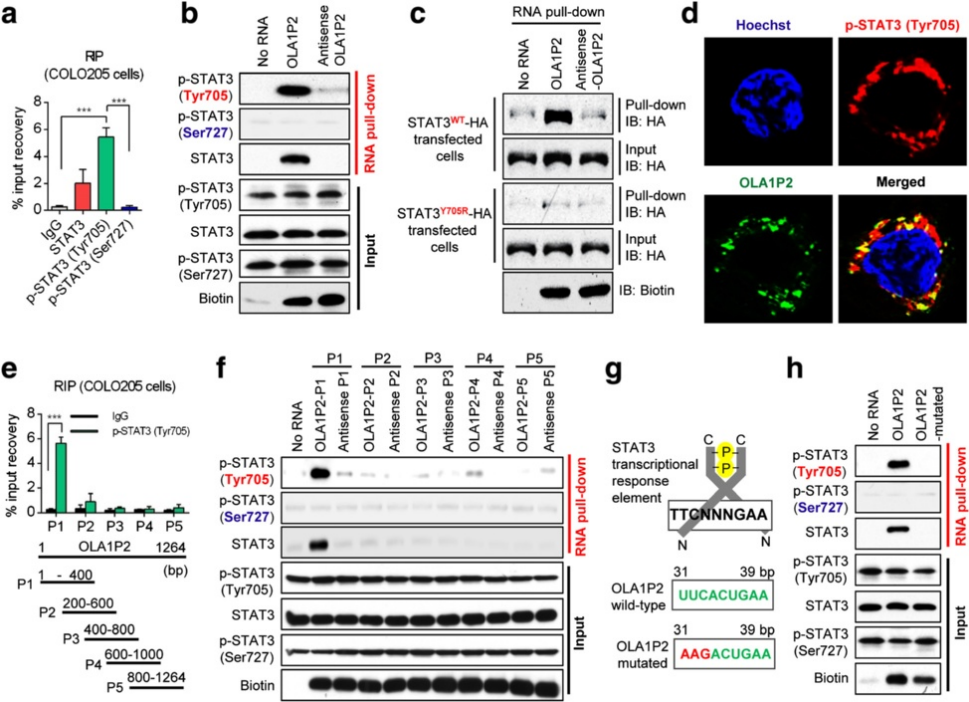
OLA1P2 interacted directly with phosphorylated STAT3 (Tyr705). **a** RIP analysis determined the recovery of OLA1P2 in COLO205 cells using STAT3 or phosphorylated STAT3 antibodies. **b** RNA pull-down analysis determined the phosphorylated STAT3 protein-lncRNA OLA1P2 interaction in COLO205 cells using 5′ biotin-linked RNAs. **c** After cells were transfected with the STAT3 expression vector (wt/Y705R) for 48 h, RNA pull-down analysis determined the STAT3 protein-OLA1P2 interaction in COLO205 cells using 5′ biotin-linked RNAs. **d** RNA FISH technology and immunofluorescence analysis determined the co-localization of OLA1P2 and phosphorylated STAT3 (Tyr705) protein in COLO205 cells. **e** RIP analysis to determine the recovery of a portion of OLA1P2 in COLO205 cells transfected with lenti-OLA1P2 using phosphorylated STAT3 (Tyr705) antibody. **f** RNA pull-down analysis to determine the interaction of phosphorylated STAT3 protein with a portion of OLA1P2 using 5′ biotin-linked RNAs. **g** Diagram showing the conserved STAT3 transcriptional response element in part 1 of OLA1P2. **h** RNA pull-down analysis to determine the interaction of phosphorylated STAT3 with mutated OLA1P2 in COLO205 cells. ***: *P* <0.001

RNA immunoprecipitation (RIP) experiments demonstrated that endogenous OLA1P2 was markedly recovered by the phosphorylated STAT3 (Tyr705) protein (Fig. 4a; Additional file 2: Figure S4A). We performed an RNA pull-down assay using 5′ biotin-linked RNAs, and the results indicated that the phosphorylated STAT3 (Tyr705) protein, but not the phosphorylated STAT3 (Ser727) protein, could be pulled down in the OLA1P2-treated group (Fig. 4b; Additional file 2: Figure S4B). Furthermore, a point mutation at the phosphorylated site (Y705R) of the STAT3 protein abrogated the affinity of STAT3 and OLA1P2 (Fig. 4c). RNA FISH technology combined with immunofluorescence analysis confirmed the co-localization of lncRNA OLA1P2 and phosphorylated STAT3 (Tyr705) protein in the cancer cells (Fig. 4d).

To investigate the region of OLA1P2 responsible for the binding activity with phosphorylated STAT3, we designed various primers to detect different fragments of OLA1P2. Only part 1 (P1) of OLA1P2 was markedly recovered by the phosphorylated STAT3 (Tyr705) protein (Fig. 4e; Additional file 2: Figure S4C). The phosphorylated STAT3 (Tyr705) protein, but not the phosphorylated STAT3 (Ser727) protein, could be pulled down by OLA1P2-P1 (Fig. 4f; Additional file 2: Figure S4D). In further analyses, we identified an evolutionarily conserved STAT3 transcriptional response element in part 1 of OLA1P2 (Fig. 4g). When this STAT3 transcriptional response element was mutated, the phosphorylated STAT3 (Tyr705) protein could not be effectively pulled down in the OLA1P2-mutated-treated group (Fig. 4h; Additional file 2: Figure S4E).

### LncRNA OLA1P2 blocked the formation of phosphorylated STAT3 homodimers

**Fig. 5:**
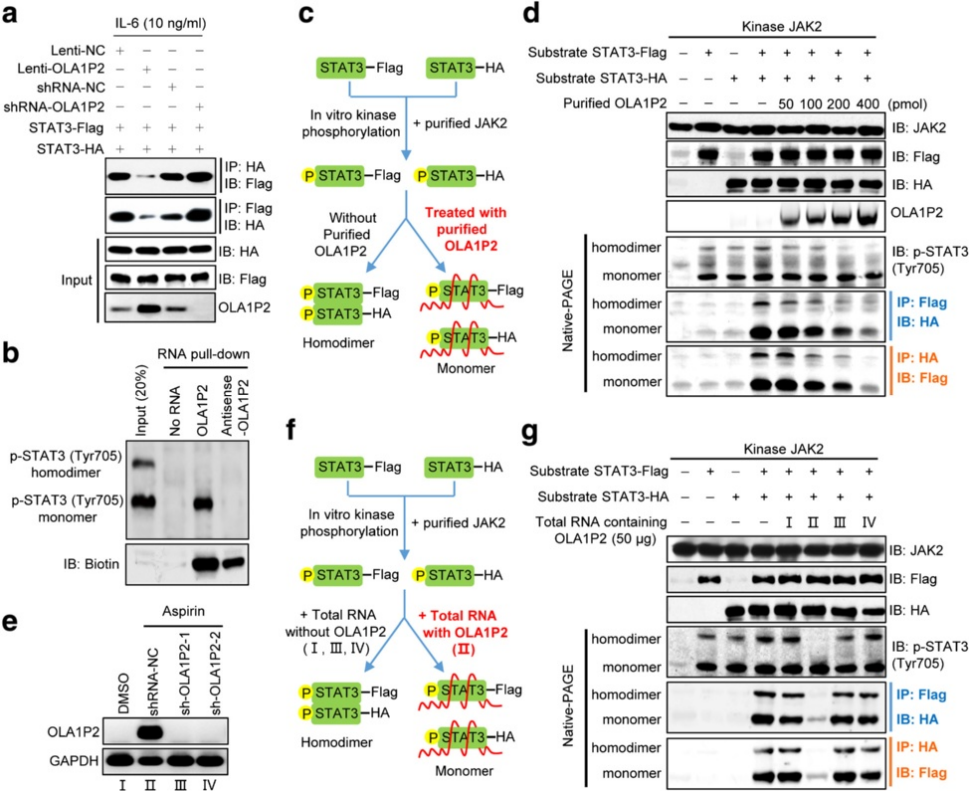
OLA1P2 blocked the formation of phosphorylated STAT3 homodimers. **a** A total of 293 T cells were transfected with indicated plasmids for 48 h and then treated with IL-6 for 24 h. Immunoprecipitation and immunoblotting analysis were performed to analyze STAT3-Flag and STAT3-HA protein levels. **b** COLO205 cells were subjected to RNA pull-down analysis using 5′ biotin-linked RNAs, and the eluted proteins were determined using immunoblotting analysis following polyacrylamide gel electrophoresis under non-denaturing conditions. **c** Schematic diagram showing the experimental design of purified OLA1P2 blocking the formation of phosphorylated STAT3 homodimers. We used 4 μg purified STAT3 (2 μg STAT3-Flag combined with 2 μg STAT3-HA) incubated with 2 μg purified JAK2. After incubation, purified OLA1P2 was then added to the reaction mixture. **d** Different amount of purified OLA1P2 blocking the formation of phosphorylated STAT3 homodimers. **e** QRT-PCR analysis of the amount of lncRNA OLA1P2 in COLO205 cells treated with aspirin and shRNA-OLA1P2. **f** Schematic diagram showing the modified experimental design of total RNA containing OLA1P2 blocking the formation of phosphorylated STAT3 homodimers. We used 4 μg purified STAT3 (2 μg STAT3-Flag combined with 2 μg STAT3-HA) incubated with 2 μg purified JAK2. After incubation, total RNA extracted from COLO205 cells were then added to the reaction mixture. **g** The total RNA of OLA1P2 silencing COLO205 cells failed to block the formation of phosphorylated STAT3 homodimers

Overexpression of OLA1P2 markedly blocked the formation of phosphorylated STAT3 homodimers, whereas silencing of OLA1P2 promoted the formation of phosphorylated STAT3 homodimers (Fig. 5a). The results of polyacrylamide gel electrophoresis under non-denaturing conditions indicated that OLA1P2 could only bind with phosphorylated STAT3 (Tyr705) monomers and did not interact with phosphorylated STAT3 (Tyr705) homodimers (Fig. 5b). We next determined whether OLA1P2 was capable of blocking the formation of phosphorylated STAT3 homodimers by conducting a competition assay. After STAT3 was phosphorylated by purified JAK2 enzymatic reactions, the variant amount of purified OLA1P2 produced by *in vitro* RNA synthesis was added to the reaction mixture (Fig. 5c). We found that OLA1P2 significantly blocked the formation of phosphorylated STAT3 (Tyr705) homodimers in the presence of 200 pmol purified OLA1P2 (Fig. 5d). Next, we modified the experimental design to determine whether the endogenous OLA1P2 in COLO205 cells could block the formation of phosphorylated STAT3 (Tyr705) homodimers *in vitro*. QRT-PCR was used to determine the amount of endogenous OLA1P2 in COLO205 cells treated with aspirin and shRNA-OLA1P2 (Fig. 5e). Total RNA extracted from COLO205 cells was used instead of purified OLA1P2 (Fig. 5f). Following *in vitro* incubation and purification, we demonstrated that 50 μg total cellular RNA containing endogenous OLA1P2 successfully blocked the formation of phosphorylated STAT3 (Tyr705) homodimers (Fig. 5g).

### OLA1P2 mediated the aspirin-induced anti-metastatic phenotype and was associated with malignant transformation in CRC

To investigate whether the transcriptional activity of the phosphorylated STAT3 (Tyr705) protein was affected by OLA1P2, we performed luciferase reporter analysis using a STAT3 reporter. Overexpression of OLA1P2 markedly suppressed luciferase activity, whereas silencing of OLA1P2 increased luciferase activity (Additional file 2: Figure S5A-D). Almost all of the STAT3 targets participated in cell growth were dramatically regulated by OLA1P2 (Additional file 2: Figure S5E-G). We further observed the anti-proliferative activity of OLA1P2 in cancer cell lines (Additional file 2: Figure S6A-C); OLA1P2 largely mediated the anti-invasive activity of aspirin in different cancer cell lines (Additional file 2: Figure S6D).

**Fig. 6:**
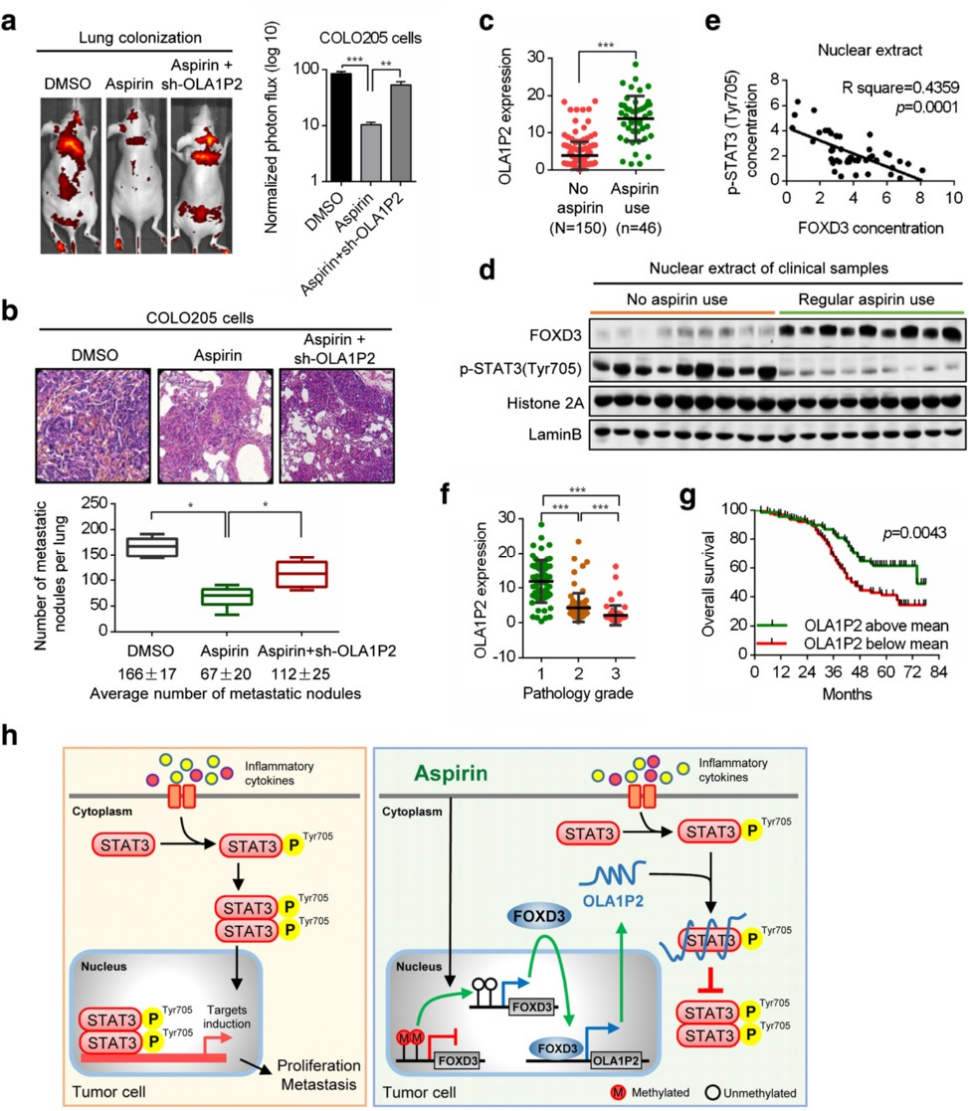
OLA1P2 mediated the aspirin-induced anti-metastatic phenotype and were associated with lower overall survival in CRC. **a** Stable OLA1P2-silencing COLO205 cells were injected into the immunodeficient mice, which were evaluated to determine the lung colonization capacity in tail vein assays. The bar graph represents 72 h time point measurements of the normalized photon flux for the animals. Representative images are shown; n = 6 for each group. **b** Number of metastases in the lungs of mice 4 weeks after tail vein injection; mean nodules per lung values are shown (bottom). Representative H&E staining of metastatic lung tumor tissues are shown (top). **c** Increased levels of OLA1P2 in clinical CRC tissues obtained from patients with regular use of aspirin were determined by qRT-PCR. **d** Immunoblotting analysis determined the protein levels of FOXD3 and phosphorylated STAT3 (Tyr705) in nuclear extract of clinical CRC samples obtained from patients with or without regular use of aspirin. **e** Linear regression analysis revealed the inverse correlation between FOXD3 and phosphorylated STAT3 (Tyr705) protein levels in nuclear extract of CRC tissues obtained from patients with regular use of aspirin (n = 46). **f** Lower OLA1P2 levels were correlated with increased pathological grade of CRC. **g** The curves show the lower overall survival of CRC patients with low OLA1P2 levels compared with CRC patients with high OLA1P2 levels. **h** A model illustrating the putative roles of aspirin-induced OLA1P2 in controlling the formation of phosphorylated STAT3 homodimers. *: *P* <0.05; **: *P* <0.01; ***: *P* <0.001

The effects of OLA1P2 on metastasis were also evaluated *in vivo*. Immunodeficient mice were pretreated with aspirin via oral administration for 8 weeks, after which cancer cells infected with either shRNA-OLA1P2 or a negative control were injected into the tail veins of immunodeficient mice. After 72 h, we observed that OLA1P2 silencing markedly weakened the anti-metastatic activity of aspirin in the lungs (Fig. 6a; Additional file 2: Figure S7A). After 4 weeks, histological analyses confirmed that the number of metastatic nodules was markedly reduced in the lungs of mice treated with aspirin alone, and OLA1P2 silencing significantly weakened the anti-metastatic activity of aspirin (Fig. 6b; Additional file 2: Figure S7B).

Increased levels of OLA1P2 in clinical cancer tissues obtained from patients with regular use of aspirin were determined by qRT-PCR analysis (Fig. 6c; Additional file 2: Figure S8A). Upregulated FOXD3 protein levels and downregulated phosphorylated STAT3 (Tyr705) protein levels were identified by immunoblot analysis in nuclear extracts of clinical cancer samples obtained from patients who regularly used aspirin (Fig. 6d; Additional file 2: Figure S8B). We found a significant inverse correlation between FOXD3 protein levels and phosphorylated STAT3 (Tyr705) protein levels in nuclear extracts of clinical cancer samples obtained from patients who regularly used aspirin (Fig. 6e; Additional file 2: Figure S8C). Lower levels of OLA1P2 correlated with more advanced pathology grade, suggesting an association between OLA1P2 expression and tumor progression (Fig. 6f; Additional file 2: Figure S9A, B). Furthermore, OLA1P2 levels below the mean correlated with lower overall survival of patients with CRC (Fig. 6g; Additional file 2: Figure S9C, D). In addition, we determined the expression of FOXD3 protein and phosphorylated STAT3 (Tyr705) protein in clinical samples using immunohistochemical analysis (Additional file 2: Figure S9E, G). Negligible and weak FOXD3 expression correlated with lower overall survival of patients with CRC (Additional file 2: Figure S9F). Moderate and strong phosphorylated STAT3 (Tyr705) expression correlated with lower overall survival of patients with CRC (Additional file 2: Figure S9H).

In conclusion, regular use of aspirin induced demethylation of the *FOXD3* promoter and promoted expression of the *FOXD3* gene. Upregulated FOXD3 protein transcriptionally activated lncRNA OLA1P2 expression. Then, OLA1P2 overexpression resulted in direct binding of OLA1P2 with phosphorylated STAT3 (Tyr705) and blocked the formation of phosphorylated STAT3 homodimers (Fig. 6h).

## Discussion

A large body of evidence has indicated that lncRNAs are involved in chemopreventive effects [19, 20]. In this study, we investigated lncRNA expression profiles in response to aspirin treatment in CRC cells and identified an aspirin-induced upregulated lncRNA, OLA1P2. Importantly, lower levels of OLA1P2 correlated with more advanced pathology grade and lower overall survival of patients with CRC. Within the first 2 years after surgery for patients with CRC, the majority of residual cancer cells often undergo a period of dormancy, a stage in cancer progression in which residual disease is present but remains asymptomatic [21]. Regardless of high or low OLA1P2 expression, the survival curve did not differ within the first 2 years due to the lower probability of cancer recurrence for all patients with CRC. More than 2 years after surgery, OLA1P2 overexpression may suppress cancer cell proliferation and metastasis, thus resulting in a lower rate of cancer recurrence. In addition to CRC, OLA1P2 was also markedly induced by aspirin in oral cancers and gastric cancers. These results indicated that aspirin might share the same chemotherapy mechanism involving OLA1P2 induction in oral cancer, gastric cancer, and CRC. However, OLA1P2 was not found to serve as a key mediator of aspirin treatment in esophageal cancer, liver cancer, or pancreatic cancer, although clinical trials have indicated that aspirin significantly reduces the risks of these cancers [22–24]. Our research indicated that different anticancer mechanisms likely underlie the chemoprotective effects of aspirin use on digestive system cancers.

We found that aspirin induced OLA1P2 expression through FOXD3 upregulation. FOXD3 belongs to the forkhead family of transcription factors. Its role in tumorigenesis has recently attracted attention [25]. Furthermore, the FOXD3 tumor-suppressive cascade was downregulated in human cancers mainly due to DNA methylation [26]. Our results confirmed that demethylation of the *FOXD3* promoter induced by aspirin might be responsible for the overexpression of FOXD3 in CRC cells. Aspirin dramatically increased FOXD3 concentrations in the nuclei of cancer cells and effectively promoted FOXD3 interaction with OLA1P2 promoter regions, providing convincing evidence that FOXD3 is a new aspirin target that could function in the control of lncRNA transcription. In addition, given that FOXD3 is critical for the maintenance of self-renewal, survival, and pluripotency in human embryonic stem cells [27], investigating the roles of aspirin in embryonic organ development may be valuable.

Many new functions have been assigned to non-coding RNAs in the cytoplasm. A muscle-specific lncRNA, linc-MD1, displays decoy activity for two specific miRNAs (miR-133 and miR-135) and regulates the expression of MAML1 and MEF2C in a molecular circuitry affecting the differentiation program [28]. All MRE (microRNA response element)-containing transcripts in the cytoplasm, including lncRNA, transcribed pseudogenes, and even protein-coding RNA, are capable of regulating each other through ceRNA (competing endogenous RNAs) activity [29]. Our results confirmed that aspirin-induced OLA1P2 was mainly enriched in the cytoplasm. Cytoplasmic OLA1P2 affected the STAT3 signaling pathway activity by inhibiting the nuclear import of phosphorylated STAT3. The accumulation of STAT3 in the nucleus was tightly controlled. A number of factors regulate the phosphorylation status of STAT3 and alter its nuclear import-export dynamics [30]. Cao *et al.* determined that the lncRNA lnc-DC could bind directly to STAT3 in the cytoplasm, promoting STAT3 phosphorylation at tyrosine-705 by preventing STAT3 from binding to and being dephosphorylated by SHP1 [31]. We found that the phosphorylation of tyrosine-705 of STAT3 was the first step in OLA1P2 binding and that the formation of phosphorylated STAT3 homodimers was subsequently blocked by OLA1P2. OLA1P2 directly interacted with STAT3 due to OLA1P2 sharing the same conservative STAT3 transcription response element as STAT3-activated targets. Given that inhibition of STAT3 homodimers formation diminished STAT3 activity, STAT3 may represent a potential therapeutic target for OLA1P2 in cancer chemotherapy.

Our results established a new link between FOXD3, STAT3, and OLA1P2. FOXD3 is an important tumor suppressor that suppresses the growth, invasion, and metastasis of cancer cells [32]. Aberrant activation of STAT3 has been reported in many types of tumors; STAT3 plays an oncoprotein role in tumorigenesis [33]. Maintaining cellular homeostasis by balancing these counter-signaling pathways is important for cell survival. Our results showed that overexpression of FOXD3 induced by aspirin could indirectly suppress STAT3 signaling pathway activity, which is mediated by the lncRNA OLA1P2. Here, lncRNAs represent a new layer of regulatory circuitry in which different types of cell components (both proteins and non-coding RNAs) can exhibit cross-talk and determine cell fates.

In this study, we investigated the lncRNA expression profiles associated with aspirin treatment and showed that lncRNA OLA1P2 could be dramatically induced by aspirin in cancer cells. Regular use of aspirin induced demethylation of the *FOXD3* promoter and promoted expression of the *FOXD3* gene. Upregulated FOXD3 protein transcriptionally activated lncRNA OLA1P2 expression. Then, OLA1P2 overexpression resulted in direct binding of OLA1P2 with phosphorylated STAT3 (Tyr705) and blocked the formation of phosphorylated STAT3 homodimers. The aspirin-FOXD3-OLA1P2-STAT3 axis showed exciting antitumor effects in relation to cancer treatment. Our research provides new insight into the chemopreventive mechanisms associated with aspirin use.

## Conclusions

We identified an aspirin-induced upregulated lncRNA, OLA1P2, in human CRC, oral cancer, and gastric cancer cells. OLA1P2 upregulation markedly affected STAT3 signaling pathway activity by inhibiting the formation of phosphorylated STAT3 homodimers. The findings of this study show that the aspirin-FOXD3-OLA1P2-STAT3 axis exhibits exciting anticancer effects and provide new insight into the chemopreventive mechanisms underlying aspirin use.

## Methods

### Patients

Between April 2007 and April 2009, we recruited 292 patients with primary GC from Shanghai Cancer Center, Fudan University. All patients (excluding stages IV disease) underwent R0 resection with D2 lymph node dissection and all patients histopathologically confirmed diagnosis of stages II and III gained chemotherapy-based fluorouracil of six to eight courses following operation. Between July 2007 and May 2010, we recruited 170 patients with oral cancer from Ninth People’s Hospital, Shanghai Jiao Tong University School of Medicine. Early-stage disease (stages I and II) was primarily managed with radical neck dissection. Advanced-stage disease (stages III and IV) underwent radical neck dissection with postoperative radiotherapy. Between September 2008 and December 2010, we recruited 196 patients with primary CRC from Ruijin Hospital, Shanghai Jiao Tong University School of Medicine. Patients underwent surgery for CRC with histological grade Dukes A, B, or C. Patients also received postsurgical adjuvant chemotherapy (Dukes C). A preoperative history of daily use of aspirin (>70 mg per day) for more than 3 years were defined as regular use of aspirin.

### Cell lines

All human cancer cell lines (SCC-9, SCC-15, SCC-25, TE-1, TE-2, TE-8, AGS, SNU-1, NCI-N87, COLO205, LoVo, SW620, HepG2, Hep3B, SMMC7721, PANC-1, SW1990, and Capan-1) were obtained from the American Type Culture Collection (Manassas, VA, USA).

### LncRNA microarray analysis

LncRNA expression profiles were investigated using the Agilent Human lncRNA array 8*60 K (OE Biotech, Shanghai, China) (GEO: GSE76583). The RNA samples were first reverse transcribed into cDNA, and these cDNA samples were then labeled using a Low Input Quick-Amp Labeling Kit (Agilent Technologies, Santa Clara, CA, USA). Labeled cDNA samples were used as probes to hybridize to lncRNA microarrays. After the samples were hybridized, the microarrays were scanned with an Agilent microarray scanner. Feature Extraction software (version 10.7.1.1, Agilent Technologies) was used to analyze array images to obtain raw data. GeneSpring software (version 12.5, Agilent Technologies) was employed to finish the basic analysis of the raw data. Initially, the raw data were normalized using the quantile algorithm. The probes with at least 100% of samples in any one condition out of two conditions having flags that indicate ‘Detected’ were chosen for further data analysis. Differentially expressed lncRNAs were then identified through fold change, and *P* values were calculated using t-tests. The thresholds set for up- and downregulated genes were a fold change ≥2.0 and a *P* value ≤0.05.

### Northern blot analysis

OLA1P2 levels were measured by northern blot using an Ambion Northern Max-Gly Kit (Austin, TX, USA). Total RNA was electrophoresed and siphoned to a positively charged nylon membrane (NC). RNA was then fixed to the NC membrane using UV cross-linking. Briefly, the cross-linked membrane was then prehybridized with ULTRAhyb, and RNA was detected with an OLA1P2-specific oligonucleotide probe (5′-TCAGCACTCAATTCTTGCCAA-3′) labeled with digoxigenin-ddUTP using a DIG Oligonucleotide 3′-End Labeling Kit (Roche Diagnostics, Indianapolis, IN, USA) in roller bottles.

### Biotin-promoter pull-down assay and mass spectrometry

Double-stranded OLA1P1 promoter was synthesized by PCR and labeled with biotin-14-dCTP according to the manufacturer’s instructions (19518-018, Invitrogen, Grand Island, NY, USA). Biotin-labeled DNA was diluted in 10 mM Tris-HCl, 10 mM MgCl_2_, 25 mM NaCl, and 10 % glycerol and incubated with nuclear protein extract at 4 °C for 12 h. In total, 100 μL streptavidin-linked magnetic beads (88816, Thermo Scientific, Waltham, MA USA) was used to pull down the biotinylated DNA at room temperature for 2 h. The beads-DNA-proteins were then washed with 1× binding and washing buffer (5 mM Tris-HCl, 1 M NaCl, 0.5 mM EDTA, and 0.005 % Tween 20) four times. The proteins were precipitated and diluted in 100 μL protein lysis buffer. One-shot mass spectrometry analyses were then performed to analyze the purified nuclear proteins.

### ChIP analysis

Five hundred million cells were fixed in 1 % formaldehyde for 30 min at room temperature, and then DNA was sheared to an average fragment size of 500 to 1,000 bp by sonication. Subsequently, chromatin was immunoprecipitated with biotin-labeled FOXD3 antibody. A positive control antibody (RNA polymerase II/RPII), a negative control normal mouse IgG, and GAPDH primers were used as controls to demonstrate the efficacy of the kit reagents (P-2025-48, Epigentek Group, Brooklyn, NY, USA). Purified chromatin was quantified by qRT-PCR using PowerUp™ SYBR® Green Master Mix (Life Technologies, Grand Island, NY, USA). The primer pairs used for this analysis are described in Additional file 1: Table S1. Signals obtained from the ChIP assay were divided by signals obtained from an input sample. This input sample represented the amount of chromatin used in the ChIP assay. Here, 1 % of starting chromatin was used as the input, and then a dilution factor of 100 or 6.644 cycles (log2 of 100) was subtracted from the Ct value of diluted input.

### Plasmid construction

The full-length sequences of OLA1P2 and the STAT3 gene were amplified using PCR methods (the primer pairs used for this analysis are described in Additional file 1: Table S1). The PCR products were first sub-cloned into the T vector and subsequently cloned into the lentiviral plasmid FUGW (Addgene, Cambridge, MA, USA). We used a Phusion Site-Directed Mutagenesis Kit (Life Technologies, Grand Island, NY, USA) for construction of the mutated STAT3 vector (STAT3^Y705R^-HA). The pLKO.1 (Addgene, Cambridge, MA, USA) lentiviral system was used for stable knock down of OLA1P2. The shRNA construct was made by cloning the double-stranded oligos shOLA1P2 (forward oligo: 5′-CCGG aaGUGCUUUGCCAAAGAUCAUua CTCGAG taATGATCTTTGGCAAAGCACtt TTTTTG-3′; reverse oligo: 5′-AATTCAAAAA aaGUGCUUUGCCAAAGAUCAUua CTCGAG taATGATCTTTGGCAAAGCACtt-3′) in AgeI and EcoRI sites of pLKO.1.

### Lentivirus package

The 293 T cells were cultured in Dulbecco’s modified Eagle’s medium (Gibco, Grand Island, NY USA) supplemented with 10 % (vol/vol) fetal bovine serum and transfected with 3 μg pLKO-shOLA1P2/pLenti-OLA1P2, 1 μg pCMV-VSV-G, and 3 μg pCMV-Delta8.9 using Lipofectamine 2000 reagent (Invitrogen, Grand Island, NY USA). After the cells were incubated overnight, the medium was replaced with 10 mL fresh medium. The virus-containing supernatants were collected at 48 and 72 h after transfection and then filtered using a 0.45 μm cellulose acetate filter (Sartorius, Bohemia, NY, USA).

### RNA extraction and qRT-PCR analysis

Total RNA was extracted from cancer cells using TRIzol Reagent (Invitrogen, Shanghai, China), and cDNA was synthesized using a PrimeScript RT reagent kit (Takara, Kusatsu, Shiga, Japan). A PCR analysis was performed using PowerUp™ SYBR® Green Master Mix (Life Technologies, Grand Island, NY, USA), and amplified PCR products were quantified and normalized using GAPDH as a control. The PCR cycle parameters for OLA1P2 and *FOXD3* expression were as follows: 1 cycle of 95 °C for 5 min followed by 40 cycles of 94 °C for 10 s and 65 °C for 40 s, with a final extension at 72 °C for 10 min. The primer pairs used for this analysis are described in Additional file 1: Table S1.

### Western blot analysis

Cells were harvested at the indicated times and rinsed twice with PBS. Cell extracts were prepared with lysis buffer and centrifuged at 13,000 g for 10 min at 4 °C. Protein samples (100 μg) were electrophoresed using 10 % polyacrylamide gels and transferred to PVDF membranes. After the membranes were blocked with 5 % BSA for 1 h at room temperature, they were incubated with FOXD3 antibody (ab67758, 1:1,000, Abcam, Cambridge, MA, USA), STAT3 antibody (#4904, 1:2,000, Cell Signaling, Danvers, MA, USA), p-STAT3(Tyr705) antibody (sc-8059, 1:1,000, Santa Cruz, Dallas, TX, USA), p-STAT3(Ser727) antibody (sc-293059, 1:1,000, Santa Cruz, Dallas, TX, USA), Flag antibody (ab49969, 1:3,000, Abcam, Cambridge, MA, USA) or HA antibody (ab18230, 1:3,000, Abcam, Cambridge, MA, USA) in 5 % BSA overnight at 4 °C. Secondary antibodies (1:3,000) were labeled with horseradish peroxidase (HRP). The signals were checked by autoradiography film when HRP substrate was added to the membranes.

### RNA FISH and protein immunofluorescence analysis

Cancer cells cultured on a glass coverslip were fixed in 4 % paraformaldehyde for 30 min and permeabilized with 0.1 % Triton X-100 for 30 min. The cells were washed three times with PBS and treated with pre-hybridization buffer (2× saline-sodium citrate) containing 10 % formamide. Hybridization was performed using a similar buffer, but with the addition of competitor RNA (tRNA) and competitor protein (BSA) to reduce background. The FITC-labeled OLA1P2 probe (5′-TCAGCACTCAATTCTTGCCAA-3′), which was diluted in hybridization buffer (200 μL), was deposited on a surface in a humid dark chamber. The glass coverslip was then placed face down on the drop and incubated at 37 °C for 12 h. After the cells were incubated with the OLA1P2 probe, the cells were washed five times with PBS and treated with p-STAT3 (Tyr705) antibody (sc-8059, 1:50, Santa Cruz, Dallas, TX, USA) for 2 h at 37 °C. The cells were washed five times with PBS and treated with anti-mouse fluorescent-labeled secondary antibody for 1 h at 37 °C. We continued with several rounds of washing (which also included an optional DAPI staining step) and finishing with mounting the coverslip onto a microscope slide using an anti-fade mounting medium.

### Cytoplasmic and nuclear RNA isolation

Cytoplasmic and nuclear RNA was extracted using Thermo Fisher BioReagents (Thermo Fisher, Grand Island, NY, USA) according to the manufacturer’s instructions. QRT-PCR analysis was performed using PowerUp™ SYBR® Green Master Mix (Life Technologies, Grand Island, NY, USA) to amplify the localization of OLA1P2 assay. The qRT-PCR conditions were as follows: 1 cycle of 95 °C for 5 min followed by 40 cycles of 94 °C for 10 s and 65 °C for 40 s, with a final extension at 72 °C for 10 min. The primer pairs used for this analysis are described in Additional file 1: Table S1.

### RNA immunoprecipitation analysis

Cancer cells were harvested by trypsinization and mechanically sheared using a homogenizer. Biotin-labeled STAT3 and phosphorylated STAT3 antibodies were added to the cell extract and incubated overnight at 4 °C. Streptavidin-coated magnetic beads were then added and incubated for 4 h at 4 °C. Magnetic beads were pelleted, washed, and re-suspended in 1 mL TRIzol. The isolated RNA was reverse transcribed to cDNA and then analyzed by qRT-PCR. The PCR cycle parameters for OLA1P2 enrichment were as follows: 1 cycle of 95 °C for 5 min followed by 40 cycles of 94 °C for 10 s and 65 °C for 40 s, with a final extension at 72 °C for 10 min. The primer pairs used for this analysis are described in Additional file 1: Table S1.

### RNA pull-down analysis

The biotinylated OLA1P2, antisense OLA1P2, or mutated OLA1P2 was mixed with proteins obtained from cancer cells for overnight at 4 °C. The complex of biotinylated lncRNA and proteins was purified using streptavidin-agarose for 4 h at 4 °C. The proteins are then eluted from the RNA-protein complex and detected by western blotting analysis.

### LncRNA-protein binding assay *in vivo*

The purified JAK2 protein from 293 T cells was washed three times with kinase assay buffer (10 mM HEPES [pH 7.4], 50 mM NaCl, 5 mM MgCl_2_, 5 mM MnCl_2_, 50 mM NaF, and 0.003 mM Na_3_VO_4_). The JAK2 protein (2 μg) was then incubated with the purified STAT3 protein (4 μg) and 250 μM adenosine triphosphate (ATP). Purified STAT3 protein was used as a substrate for each assay. The reaction system was incubated for 120 min at room temperature. After incubation, serial dilutions of purified lncRNA OLA1P2 or total RNA extracted from COLO205 cells were then added to the reaction mixture at room temperature for 120 min. Each reaction was then divided into three parts: the first part was used as an input, the second part was used for immunoprecipitation assay with Flag beads, and the third part was used for immunoprecipitation assay with HA beads. Eluted proteins were then subjected to polyacrylamide gel electrophoresis under non-denaturing conditions.

### *In vivo* analysis

Immunodeficient mice were pretreated with aspirin via oral administration (40 mg/kg) for 8 weeks, after which cancer cells (1 × 10^6^) stably silencing OLA1P2 were injected into the tail veins of immunodeficient mice (n = 6). Xenografts were then treated with aspirin at a dose of 40 mg/kg, administered daily via oral gavage. After 72 h, the lung colonization capacity of the cancer cells was analyzed based on the normalized photon flux.

### Hematoxylin and eosin staining

Mouse tumor tissues were fixed with formalin, embedded with paraffin, spliced into 6-μm sections, deparaffinized with xylene, and submerged into EDTA antigenic retrieval buffer for antigenic retrieval. Lung sections were stained with hematoxylin-eosin. Digital images of organs were acquired by Nanozoomer (Hamamatsu Photonics).

### Immunohistochemical staining

For immunohistochemical staining, tissues sections were incubated at 4 °C overnight with FOXD3 antibody (ab67758, 1:200, Abcam, Cambridge, MA, USA) or phosphorylated STAT3 (Tyr705) antibody (sc-8059, 1:200, Santa Cruz, Dallas, TX, USA). Sections were then rinsed in PBS-T (PBS containing 0.05 % Triton X-100), and secondary antibody was then added at a 1:500 dilution at room temperature for 1 h. After washing twice with PBS-T, the slides were incubated with streptavidin-horseradish peroxidase and diaminobenzidine substrate for colorimetric development.

### Statistical analyses

All data were expressed as mean ± standard deviation. Two variables of microarray data and qRT-PCR data were analyzed using Student’s t-test. The 5-year survival rate analyses of cancer patients were performed using log-rank (Mantel–Cox) test. A *P* value of <0.05 was considered significant.

### Ethics

The Ethics Committee of Shanghai Jiao Tong University approved our study. All participants provided written informed consent prior to enrollment. All experimental methods complied with the Helsinki Declaration. All animal studies have been approved by the Shanghai Jiao Tong University Institute Animal Care and Use Committee, and all mice were kept in the Shanghai Jiao Tong University School of medicine animal facilities.

### Data availability

Our Agilent Human LncRNA microarray data have been approved and assigned GEO accession numbers as GSE76583. You may view the GSE76583 study at: http://130.14.29.110/geo/query/acc.cgi?acc=GSE76583.

## Additional files

Additional file 1:
**Clinicopathological characteristics of cancer patients used in this study.** **Table S1.** All primer pairs used in this study. **Table S2.** Clinicopathological characteristics of 196 CRC patients. **Table S3.** Clinicopathological characteristics of 292 GC patients. **Table S4.** Clinicopathological characteristics of 170 oral cancer patients. (DOCX 34 kb) Additional file 2:
**All supplementary figures associated with lncRNA OLA1P2.** **Figure S1.** Dysregulation of genes in primary cultured colon cancer cells transfected with shRNA-OLA1P2. **Figure S2.** OLA1P2 affected STAT3 targets expression. **Figure S3.** OLA1P2 affected the translocation of the phosphorylated STAT3 protein. **Figure S4.** OLA1P2 interacted directly with phosphorylated STAT3 (Tyr705). **Figure S5.** The transcriptional activity of the phosphorylated STAT3 (Tyr705) protein was affected by OLA1P2. **Figure S6.** OLA1P2 suppressed cancer cells proliferation and mediated the aspirin-induced anti-invasive phenotype. **Figure S7.** OLA1P2 mediated the aspirin-induced anti-metastatic phonotype. **Figure S8.** The expression levels of OLA1P2, FOXD3, and phosphorylated STAT3 (Tyr705) in clinical tumor tissues. **Figure S9.** Clinical pathological features correlation analysis. (PDF 10719 kb)

## Abbreviations

BSA: bovine serum albumin
CHIP: chromatin immunoprecipitation
DMEM: Dulbecco’s modified Eagle medium
FBS: fetal bovine serum
FISH: fluorescent *in situ* hybridization
FOXD3: forkhead box D3
IB: immunoblot
IF: immunofluorescence
IP: immunopurification
PBS: phosphate buffered saline
RIP: RNA immunoprecipitation
STAT3: signal transducer and activator of transcription 3
WT: wild type
Y705R: Tyrosine 705 Arginine
