# Supplementary material for: The aspirin-induced long non-coding RNA OLA1P2 blocks phosphorylated STAT3 homodimer formation
Source: Genome Biol. 2016 Feb 22;17:24. doi: 10.1186/s13059-016-0892-5 (PMC4762163; doi:10.1186/s13059-016-0892-5)
Supplement: Additional file 1: — Clinicopathological characteristics of cancer patients used in this study. Table S1. All primer pairs used in this study. Table S2. Clinicopathological characteristics of 196 CRC patients. Table S3. Clinicopathological characteristics of 292 GC patients. Table S4. Clinicopathological characteristics of 170 oral cancer patients. (DOCX 34 kb) [file 13059_2016_892_MOESM1_ESM.docx]

**Aspirin-induced long non-coding RNA OLA1P2 blocks phosphorylated STAT3 homodimer formation**

**Supplementary Table S1. All primer pairs used in this study.**

| **Name** | **Pairs** | **Sequence (5'-3')** | **Note** |
| --- | --- | --- | --- |
| OLA1P2-Vector | Sense | CAATCATTGGAAGATTTGGAA | For expression vector construction |
|  | Antisense | CTTTCTTCTTCGGTTGTTGAG |  |
| OLA1P2-RT | Sense | ACTTCCTATTTGGCACCATTGAT | For qRT-PCR  analysis |
|  | Antisense | CAAGGCCAGCAGTATCCACT |  |
| OLA1P2-NB | Probe | AATTAAATAGTGGGTGGACAA | For northern blotting analysis (Figure1D) |
| OLA1P2 promoter-P1 | Sense | ACAAGTTCTGGTTCATGGATCT | For CHIP analysis  (Figure 2G) |
|  | Antisense | TCTTCTCTTGAGCAAACAGCTC |  |
| OLA1P2 promoter-P2 | Sense | AATGGCTGTAAGAGGCAGCAA |  |
|  | Antisense | CAAACTGAGGGGGAAAAAGCAG |  |
| OLA1P2 promoter-P3 | Sense | TCCTCCCACAATGGGAAATGA |  |
|  | Antisense | ACTGTAGATGGCCAAGCATCA |  |
| OLA1P2 promoter-P4 | Sense | CGAAAGCTACGAAACTCACAGC |  |
|  | Antisense | AGGACAGCAACTGCTGCTAA |  |
| OLA1P2 promoter-P5 | Sense | TAGGACCTTTCTCTCGCAGC |  |
|  | Antisense | TTCCATCACCTCCCTCTTTGC |  |
| OLA1P2-P1 | Sense | TGGTGGATTGCTAAATGTTGGA | For RIP analysis  (Figure 4E) |
|  | Antisense | GGATCAATGGTGCCAAATAGG |  |
| OLA1P2-P2 | Sense | ATGGTGATATGACGCATGTTGA |  |
|  | Antisense | ATGGACCCAATCATTTCCTCCT |  |
| OLA1P2-P3 | Sense | AGGAGGAAATGATTGGGTCCAT |  |
|  | Antisense | ATCTCCTCCTCTCACAGCCA |  |
| OLA1P2-P4 | Sense | CTAGGTGCCTTTGTCGTTCCT |  |
|  | Antisense | TTGCTTCCAGTTACTCCGGT |  |
| OLA1P2-P5 | Sense | GACCTAGGTGCCTTTGTCGT |  |
|  | Antisense | GCTGCAGACCCAGCCTTAAT |  |
| FOXD3-RT | Sense | GACATGTTCGACAACGGCAG | For qRT-PCR analysis  (Figure 2E) |
|  | Antisense | CTGTAAGCGCCGAAGCTCTG |  |
| FOXD3-Vector | Sense | ATGACCCTCTCCGGCGGC | For expression vector construction |
|  | Antisense | CTATTGCGCCGGCCATTTGGCTT |  |
| Methylated-specific primer | Sense | GTTAGTTTTTAAAACGGGATTTT | For FOXD3 promoter methylation analysis  (Figure 2E) |
|  | Antisense | CTCGACCTCTAACCCTAACGT |  |
| Unmethylated-specific primer | Sense | TTAGTTTTTAAAATGGGATTTTTGA |  |
|  | Antisense | TTCCTCAACCTCTAACCCTAACATA |  |
| STAT3-Vector | Sense | TTGGCTGAAGGGGCTGTAAT | For expression vector construction |
|  | Antisense | GTAGGCGCCTCAGTCGTATC |  |
| GAPDH | Sense | CACTAGGCGCTCACTGTTCTC | For qRT-PCR  analysis |
|  | Antisense | AAATCCGTTGACTCCGACCT |  |
| U2 | Sense | GGCCTTTTGGCTAAGATCAA | For qRT-PCR  analysis |
|  | Antisense | TATTCCATCTCCCTGCTCCA |  |

**Supplementary Table S2. Clinicopathological characteristics of 196 CRC patients.**

| **Clinicopathological features** | **Cases (n)** | **%** | **OLA1P2** | | ***P* value** |
| --- | --- | --- | --- | --- | --- |
|  |  |  | **Upregulation (above mean)** | **Downregulation (below mean)** |  |
| Age (years) |  |  |  |  | 0.9898 |
| ≥60 | 131 | 66.84 | 45 | 86 |  |
| <60 | 65 | 33.16 | 23 | 42 |  |
| Gender |  |  |  |  | 0.9917 |
| Male | 105 | 53.57 | 36 | 69 |  |
| Female | 91 | 46.43 | 32 | 59 |  |
| Tumor size (cm) |  |  |  |  | <0.0001 |
| ≥5 | 114 | 58.16 | 12 | 102 |  |
| <5 | 82 | 41.84 | 56 | 26 |  |
| TNM stage |  |  |  |  | <0.0001 |
| Stage 1/2 | 118 | 60.20 | 61 | 57 |  |
| Stage 3/4 | 78 | 39.80 | 7 | 71 |  |
| Pathologic grade |  |  |  |  | <0.0001 |
| N1 | 63 | 32.14 | 46 | 17 |  |
| N2 | 78 | 39.80 | 19 | 59 |  |
| N3 | 55 | 28.06 | 3 | 52 |  |
| Lymph node status |  |  |  |  | <0.0001 |
| No metastasis | 124 | 63.27 | 63 | 61 |  |
| Metastasis | 72 | 36.73 | 5 | 67 |  |

**Supplementary Table S3. Clinicopathological characteristics of 292 GC patients.**

| **Clinicopathological features** | **Cases (n)** | **%** | **OLA1P2** | | ***P* value** |
| --- | --- | --- | --- | --- | --- |
|  |  |  | **Upregulation (above mean)** | **Downregulation (below mean)** |  |
| Age (years) |  |  |  |  | 0.9997 |
| ≥60 | 176 | 60.27 | 65 | 111 |  |
| <60 | 116 | 39.73 | 43 | 73 |  |
| Gender |  |  |  |  | 0.9722 |
| Male | 184 | 63.01 | 69 | 115 |  |
| Female | 108 | 36.99 | 39 | 69 |  |
| Tumor size (cm) |  |  |  |  | <0.0001 |
| ≥5 | 164 | 56.16 | 22 | 142 |  |
| <5 | 128 | 43.84 | 86 | 42 |  |
| Site |  |  |  |  | 0.9998 |
| Cardia-fundus | 137 | 46.92 | 51 | 86 |  |
| Corpus | 61 | 20.89 | 22 | 39 |  |
| Antrum | 65 | 22.26 | 24 | 41 |  |
| Two or more sites | 29 | 9.93 | 11 | 18 |  |
| TNM stage |  |  |  |  | <0.0001 |
| T1 | 15 | 5.14 | 14 | 1 |  |
| T2 | 60 | 20.55 | 56 | 4 |  |
| T3 | 92 | 31.51 | 36 | 56 |  |
| T4 | 125 | 42.81 | 2 | 123 |  |
| Pathologic grade |  |  |  |  | <0.0001 |
| N1 | 69 | 23.63 | 51 | 18 |  |
| N2 | 101 | 34.59 | 47 | 54 |  |
| N3 | 122 | 41.78 | 10 | 112 |  |
| Intravascular embolus |  |  |  |  | 0.9913 |
| No | 215 | 73.63 | 80 | 135 |  |
| Yes | 77 | 26.37 | 28 | 49 |  |

**Supplementary Table S4. Clinicopathological characteristics of 170 oral cancer patients.**

| **Clinicopathological features** | **Cases (n)** | **%** | **OLA1P2** | | ***P* value** |
| --- | --- | --- | --- | --- | --- |
|  |  |  | **Upregulation (above mean)** | **Downregulation (below mean)** |  |
| Age (years) |  |  |  |  | 0.9995 |
| ≥60 | 75 | 44.12 | 18 | 57 |  |
| <60 | 95 | 55.88 | 23 | 72 |  |
| Gender |  |  |  |  | 0.9954 |
| Male | 113 | 66.47 | 27 | 86 |  |
| Female | 57 | 33.53 | 14 | 43 |  |
| Pathologic grade |  |  |  |  | <0.0001 |
| N1 | 51 | 30.00 | 25 | 26 |  |
| N2 | 56 | 32.94 | 14 | 42 |  |
| N3 | 63 | 37.06 | 2 | 61 |  |
| TNM stage |  |  |  |  | <0.0001 |
| Stage ½ | 74 | 43.53 | 35 | 39 |  |
| Stage ¾ | 96 | 56.47 | 6 | 90 |  |
| Site |  |  |  |  | 0.9998 |
| Tongue | 83 | 48.82 | 20 | 63 |  |
| Gingival | 34 | 20.00 | 8 | 26 |  |
| Cheek | 19 | 11.18 | 5 | 14 |  |
| Floor of mouth | 29 | 17.06 | 7 | 22 |  |
| Oropharynx | 5 | 2.94 | 1 | 4 |  |
| Histologic signs of severity  (vascular emboli,  perineural invasion,  diffuse infiltration) |  |  |  |  | <0.0001 |
| None | 96 | 56.47 | 36 | 60 |  |
| Presence | 69 | 40.59 | 4 | 65 |  |
| Missing | 5 | 2.94 | 1 | 4 |  |
| Smoking history |  |  |  |  | 0.9967 |
| Non-smoker | 71 | 41.76 | 17 | 54 |  |
| Smoker | 94 | 55.29 | 23 | 71 |  |
| Missing | 5 | 2.94 | 1 | 4 |  |
| Alcohol history |  |  |  |  | 0.9973 |
| Non-drinker | 95 | 55.88 | 23 | 72 |  |
| Drinker | 70 | 41.18 | 17 | 53 |  |
| Missing | 5 | 2.94 | 1 | 4 |  |
