# Supplementary material for: The aspirin-induced long non-coding RNA OLA1P2 blocks phosphorylated STAT3 homodimer formation
Source: Genome Biol. 2016 Feb 22;17:24. doi: 10.1186/s13059-016-0892-5 (PMC4762163; doi:10.1186/s13059-016-0892-5)
Supplement: Additional file 2: — All supplementary figures associated with lncRNA OLA1P2. Figure S1. Dysregulation of genes in primary cultured colon cancer cells transfected with shRNA-OLA1P2. Figure S2. OLA1P2 affected STAT3 targets expression. Figure S3. OLA1P2 affected the translocation of the phosphorylated STAT3 protein. Figure S4. OLA1P2 interacted directly with phosphorylated STAT3 (Tyr705). Figure S5. The transcriptional activity of the phosphorylated STAT3 (Tyr705) protein was affected by OLA1P2. Figure S6. OLA1P2 suppressed cancer cells proliferation and mediated the aspirin-induced anti-invasive phenotype. Figure S7. OLA1P2 mediated the aspirin-induced anti-metastatic phonotype. Figure S8. The expression levels of OLA1P2, FOXD3, and phosphorylated STAT3 (Tyr705) in clinical tumor tissues. Figure S9. Clinical pathological features correlation analysis. (PDF 10719 kb) [file 13059_2016_892_MOESM2_ESM.pdf]

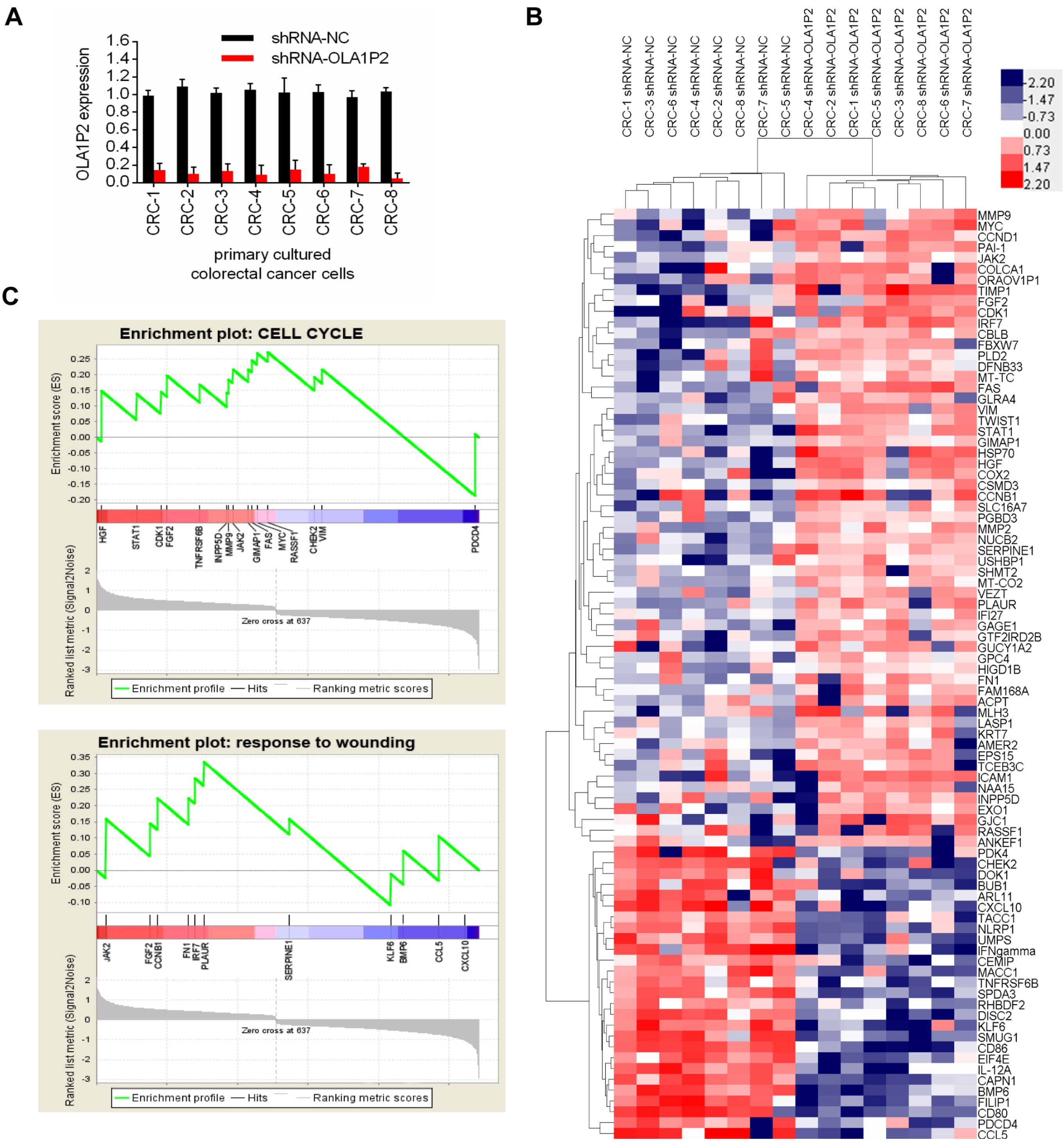

Supplementary Figure S1

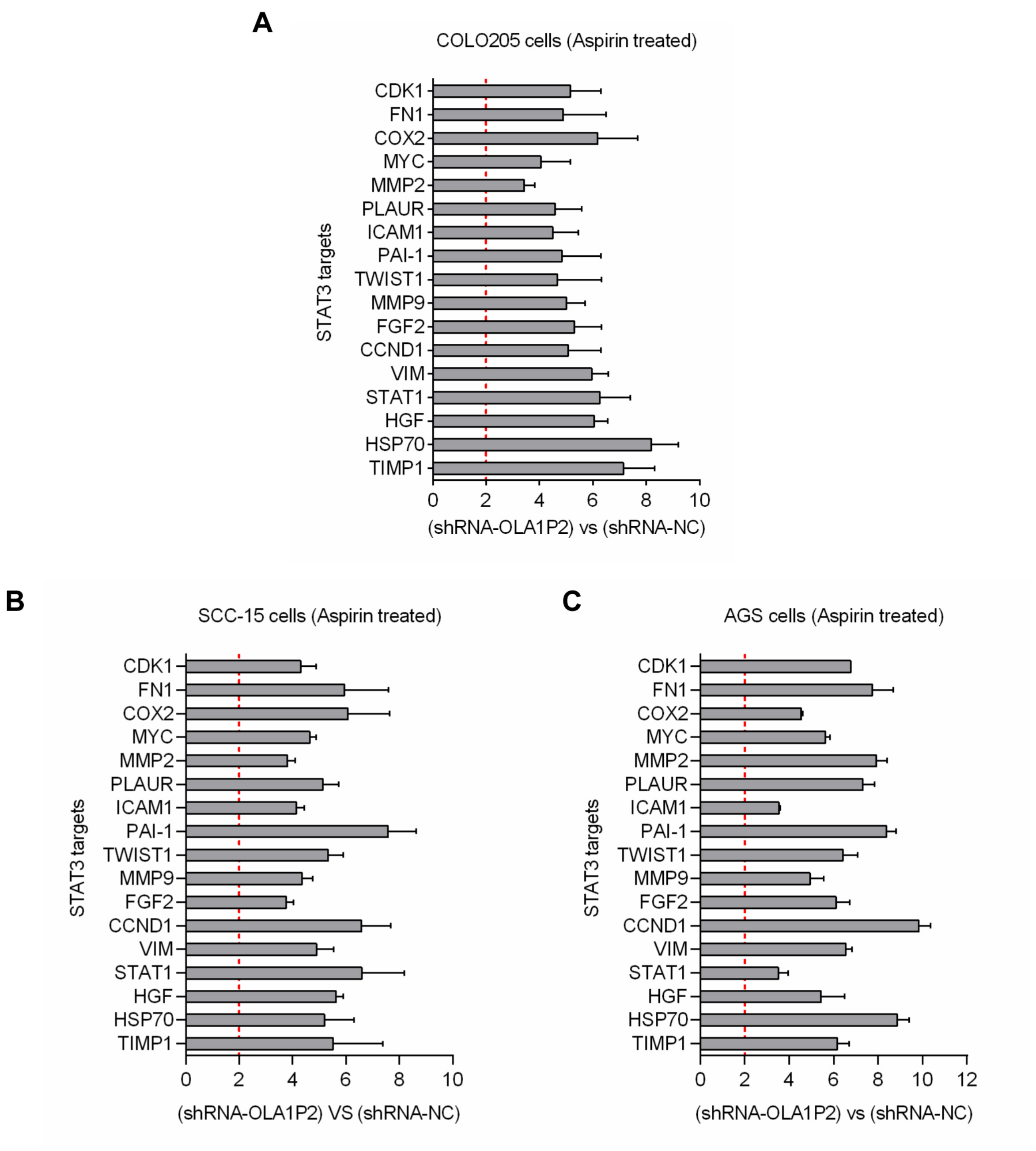

Supplementary Figure S2

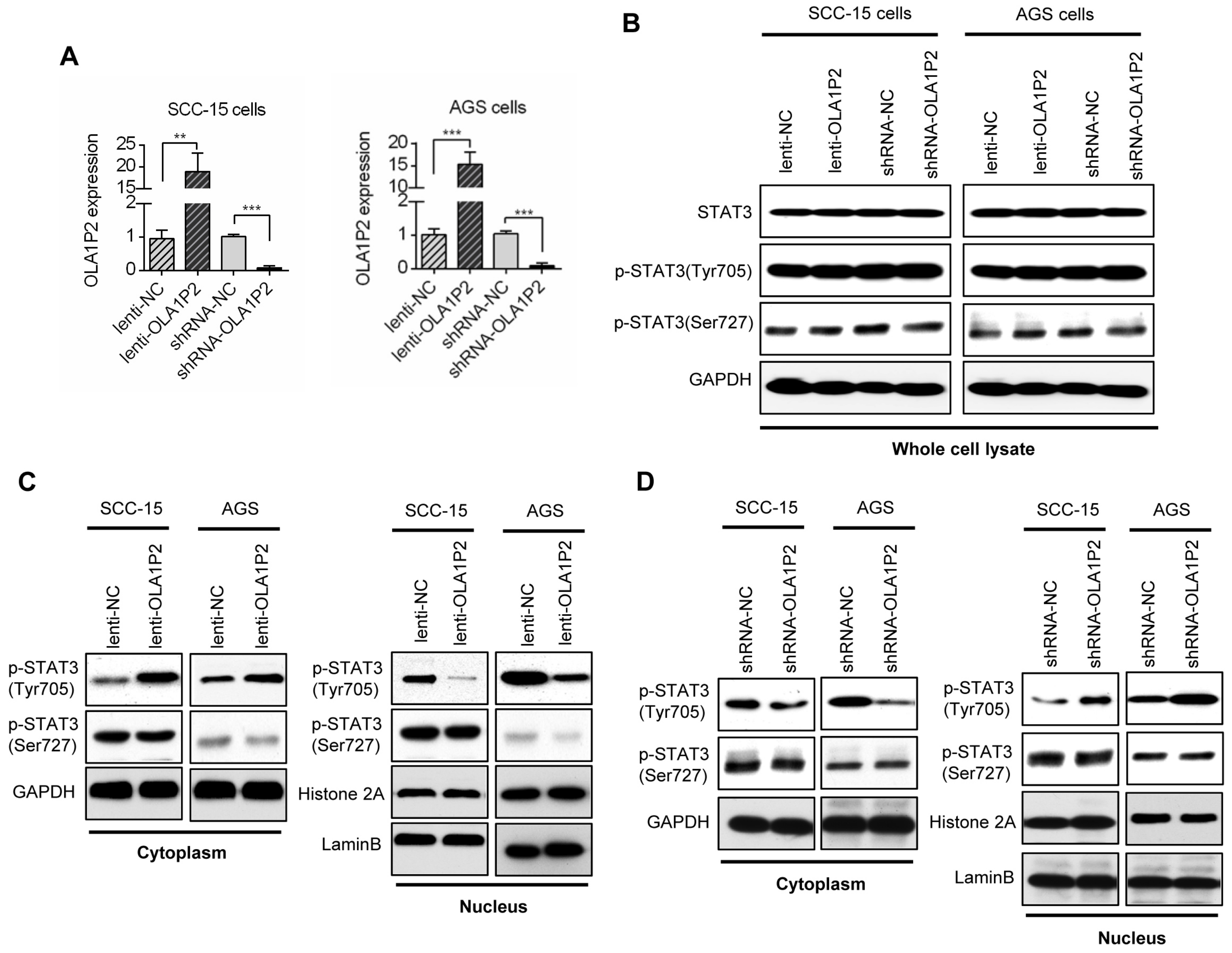

Supplementary Figure S3

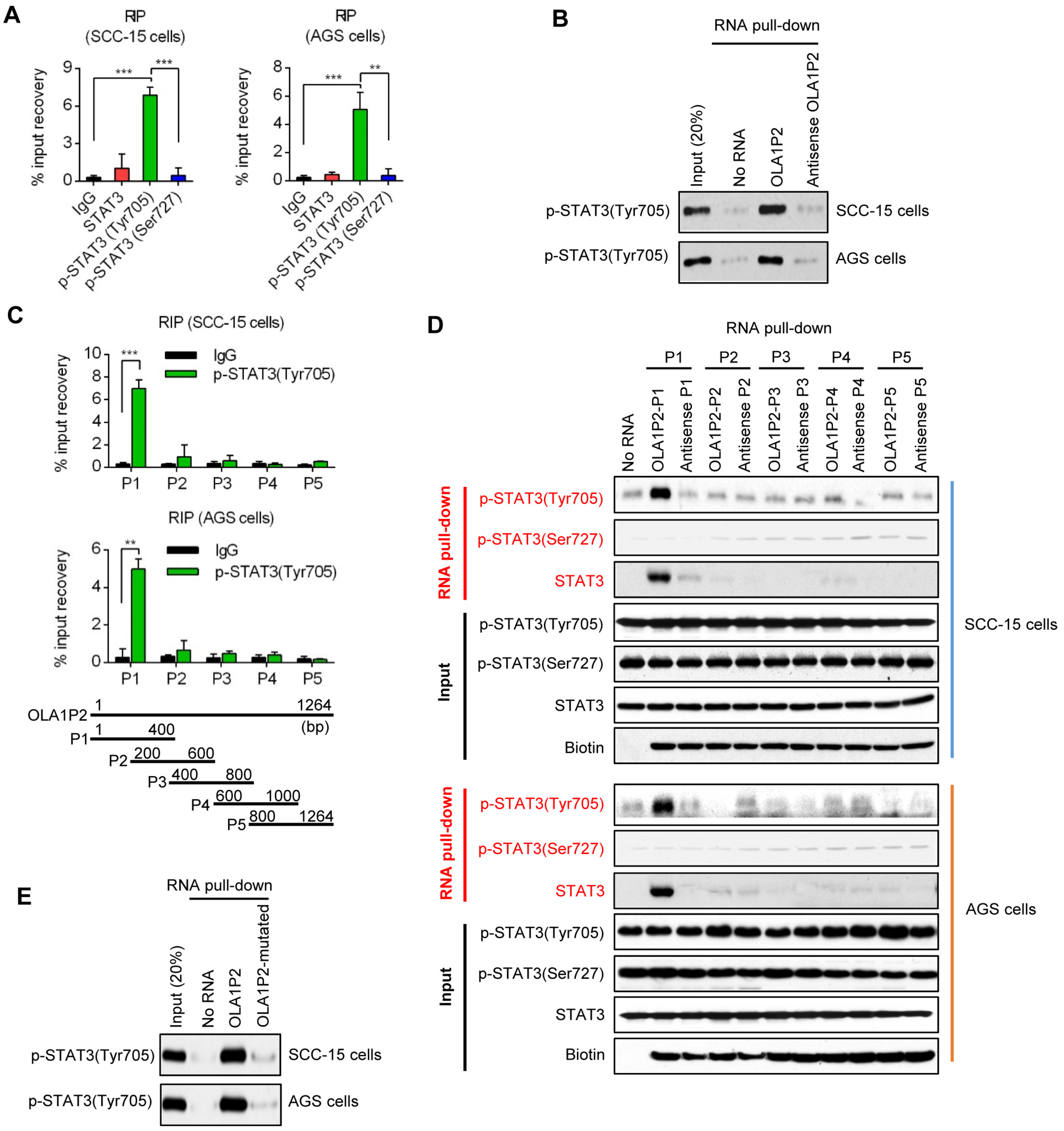

Supplementary Figure S4

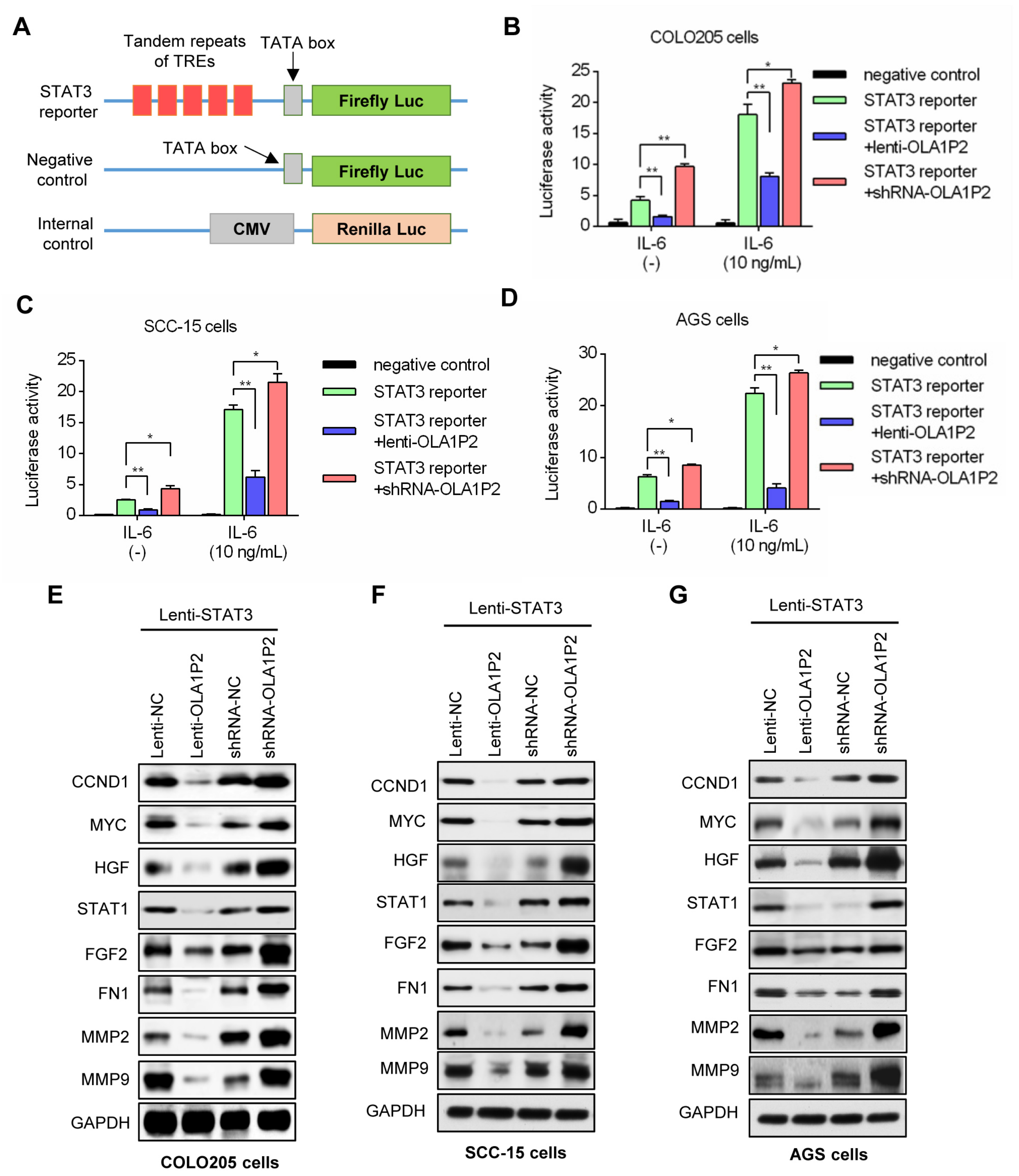

Supplementary Figure S5

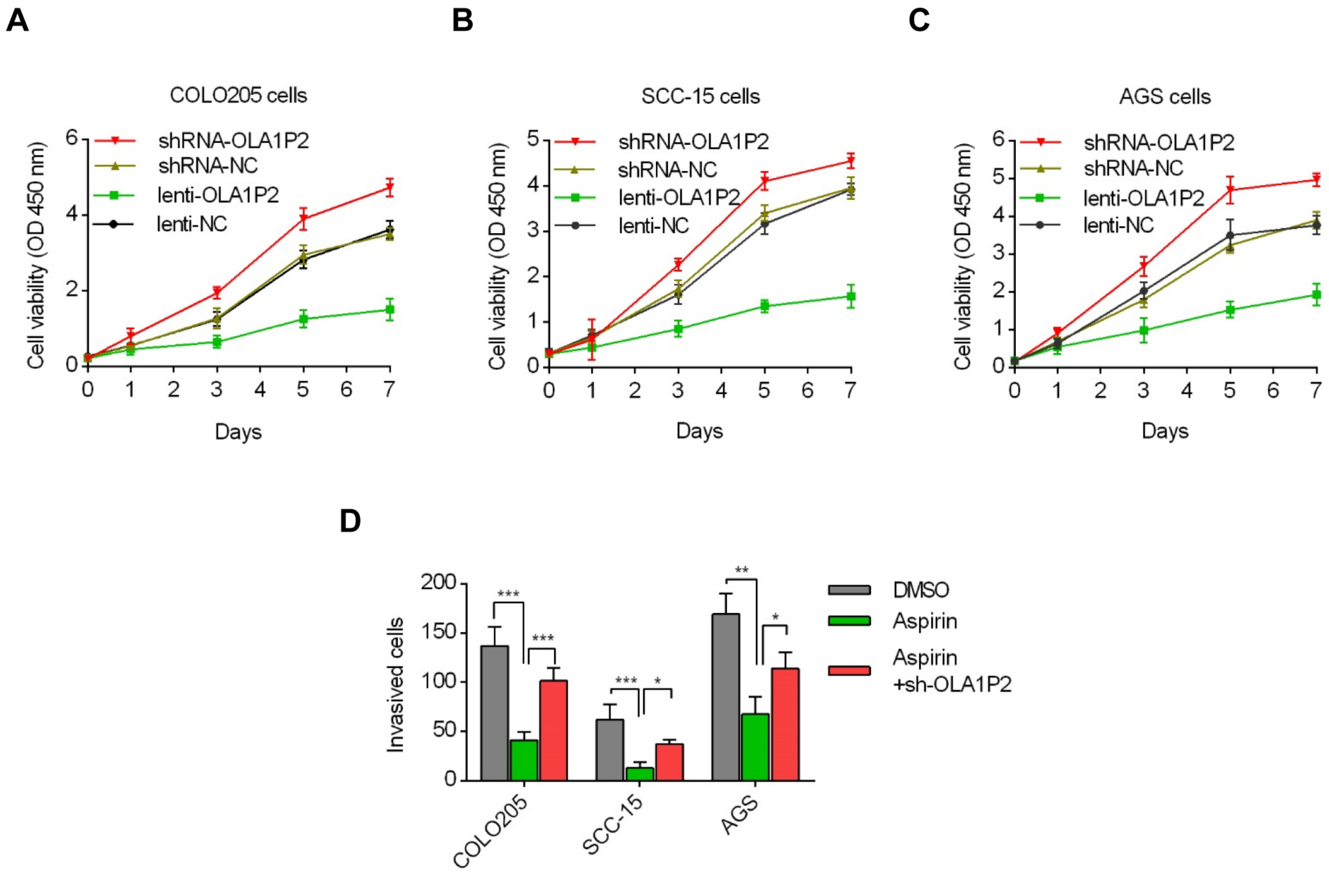

Supplementary Figure S6

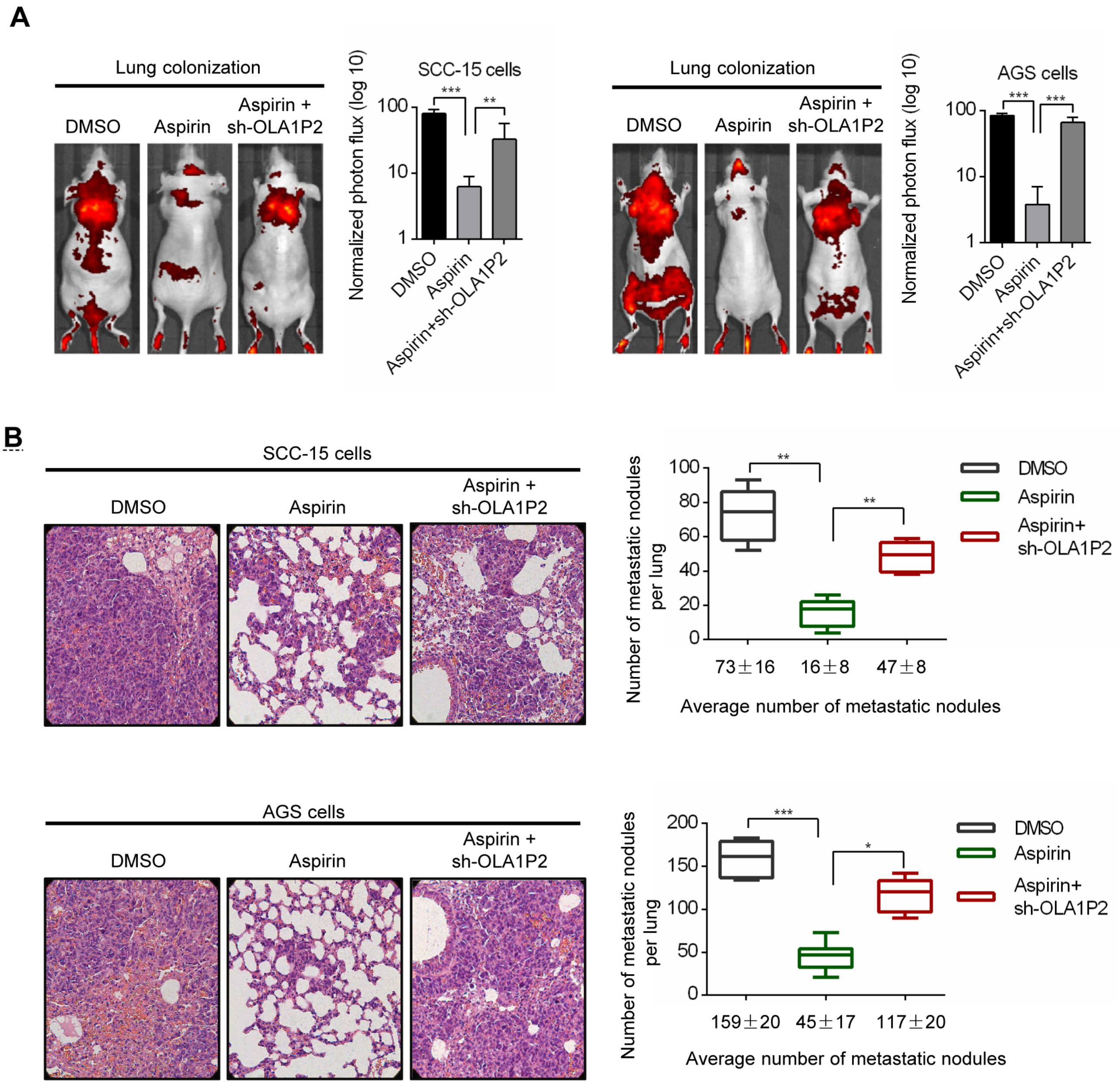

Supplementary Figure S7

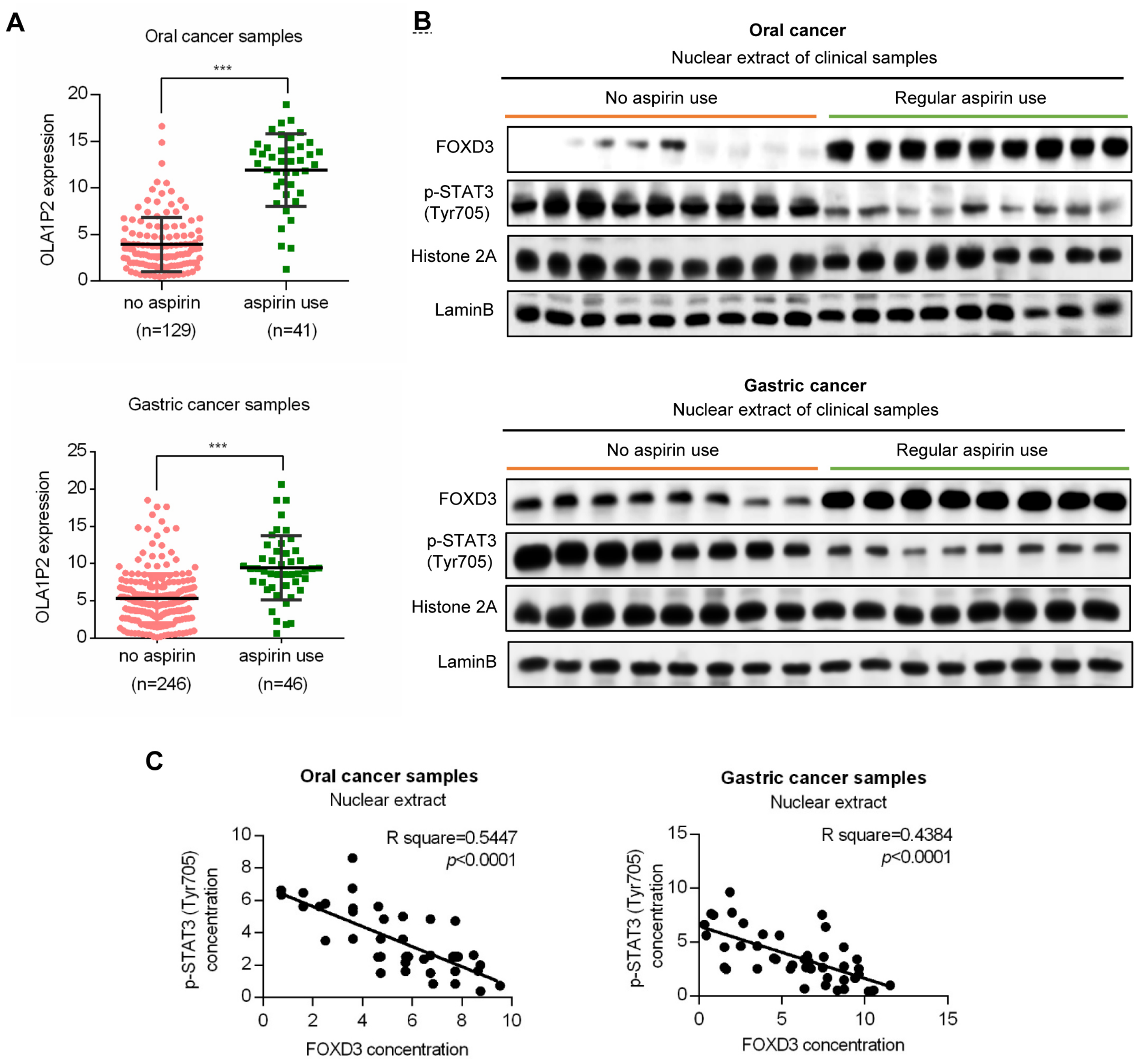

Supplementary Figure S8

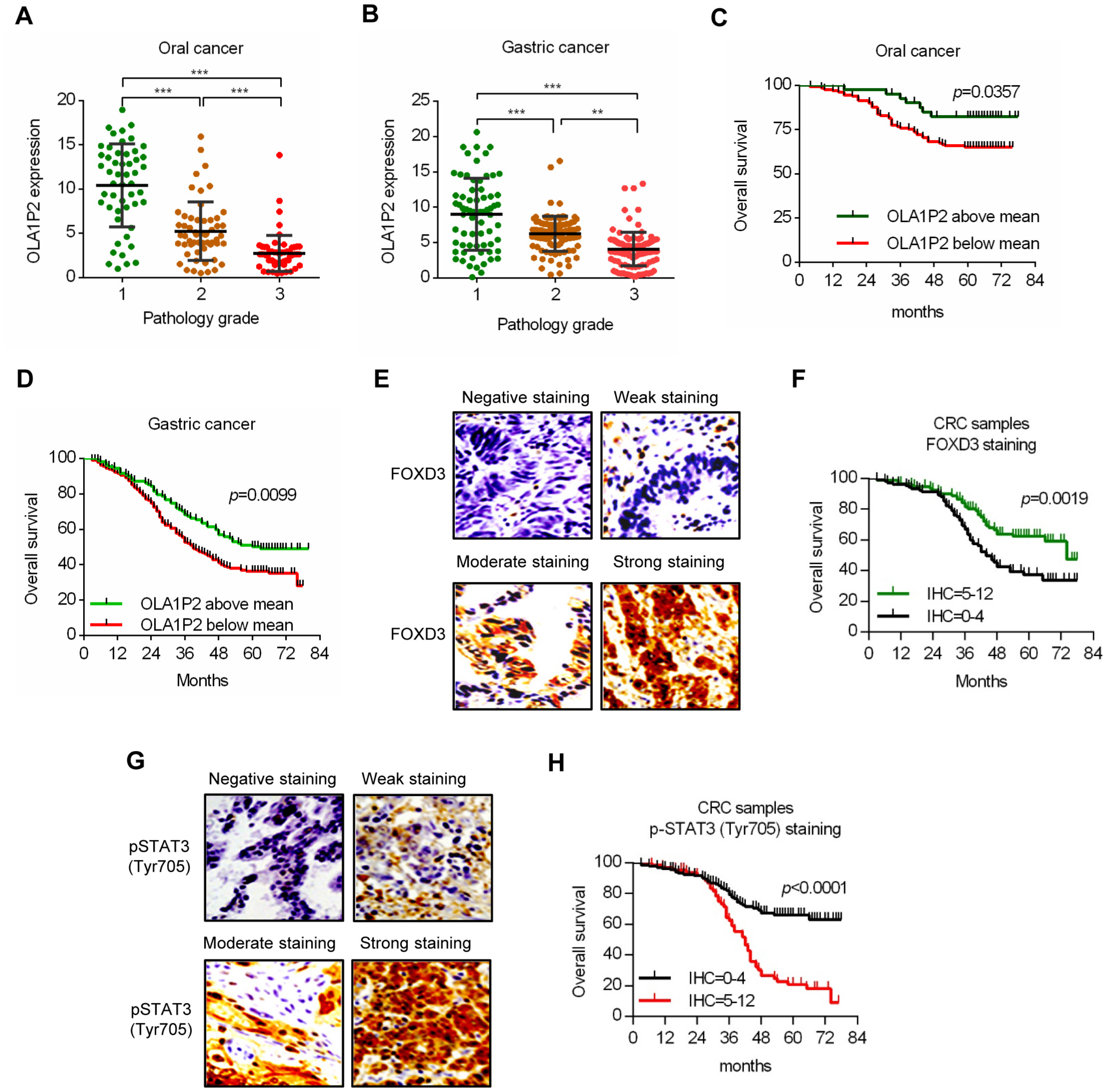

Supplementary Figure S9
